# Prioritization of Physio-Biochemical Selection Indices and Yield-Attributing Traits toward the Acquisition of Drought Tolerance in Chickpea (*Cicer arietinum* L.)

**DOI:** 10.3390/plants12183175

**Published:** 2023-09-05

**Authors:** Prakash N. Tiwari, Sharad Tiwari, Swapnil Sapre, Niraj Tripathi, Devendra K. Payasi, Mrinalini Singh, Satyendra Thakur, Mohini Sharma, Sushma Tiwari, Manoj Kumar Tripathi

**Affiliations:** 1 Biotechnology Centre, Jawaharlal Nehru Krishi Vishwa Vidyalaya, Jabalpur 482004, India; tiwarisprakashn051194@gmail.com (P.N.T.); swapnil.spr@gmail.com (S.S.); mrinalinisingh820@gmail.com (M.S.); 2 Directorate of Research, Jawaharlal Nehru Krishi Vishwa Vidyalaya, Jabalpur 482004, India; nirajtripathi@jnkvv.org; 3 Regional Agricultural Research Station, Sagar 470001, India; dpayasi@gmail.com; 4 Department of Plant Physiology, Jawaharlal Nehru Krishi Vishwa Vidyalaya, Jabalpur 482004, India; thakursatyendrasing@gmail.com; 5 Department of Plant Molecular Biology and Biotechnology, Rajmata Vijyaraje Scindia Krishi Vishwa Vidyalaya, Gwalior 474002, India; mohinisharma504@gmail.com (M.S.); sushma2540@gmail.com (S.T.)

**Keywords:** chickpea, drought tolerance, selection indices, physiological, biochemical, yield-attributing traits

## Abstract

Chickpea is widely grown in rainfed areas of developing countries because of its nutritional abundance and adaptability. To overcome the environmental effect of drought on yield, a characteristic-linked selection strategy is proved as well-thought-out and advantageous for the development of drought-tolerant cultivars. To precisely understand the contribution of various physio-biochemical and yield-attributing traits toward drought tolerance in chickpea (*Cicer arietinum* L.), forty chickpea genotypes were evaluated in the years 2020–2021 and 2021–2022 under normal irrigated as well as drought-stressed conditions. Among the studied genotypes, genotype ICC4958 retained the highest chl content (0.55 mg g^−1^ FW), minimal electrolyte leakage, and superoxide dismutase (1.48 U/mg FW) and peroxidase (2.21 µmol/min/g FW) activities while cultivar JG11 maintained the maximum relative water content and proline accumulation. The principal-component-based biplots prioritized the physio-biochemical and yield-accrediting characteristics based on their association significance and contribution to terminal drought tolerance. Under drought stress, grain yield per plant was depicted to have a strongly positive association with canopy temperature depression, catalase, superoxide dismutase, and peroxidase activities as well as total soluble sugar, proline, and chlorophyll content, along with the numbers of pods and biological yield per plant. These identified physio-biochemical and yield-attributing traits can be further deployed to select drought-tolerant chickpea genotypes for the breeding of climate-smart chickpea genotypes.

## 1. Introduction

Chickpea (*Cicer arietinum* L.) is the oldest and most popular legume [1], particularly in the Mediterranean region, Asia, and America [2]. This important legume has a ridiculous basis of minerals, including calcium, phosphorus, iron, magnesium, potassium amino acids, and vitamins [3]. The seeds of chickpeas also contain carbohydrates, 59%; protein, 29%; oil, 5%; ash, 4%; and fiber, 3% [4,5]. Chickpeas are grown across 59 nations in an area of 14.8 Mha, producing 15.1 million tons annually [6]. In India, chickpeas played a major role in realizing the “Pulse Revolution” in the country by contributing more than 40% of the total pulses by area and production. The major producer of chickpeas is India, contributing 70% of the world’s area and production. Although the country has developed the ability to export kabuli-type chickpeas in the recent past, it also imports a large quantity of desi chickpeas to meet domestic demand. Desi chickpeas have the potential to be a source of different nutrients, including high-quality proteins made up of significant amounts of vital fatty acids, amino acids, minerals, trace elements, and proteins, viz., albumins and globulins [7,8]. Furthermore, there is the additional requirement of increasing chickpea production to meet the projected domestic chickpea demand of 17.5 mt by 2050 [9,10].

Chickpeas, being a traditional cool season crop of the rabi season of Northern India [11], have an indeterminate growth habit, leading to a long vegetative and reproductive period [12]. Approximately 4 million ha of area under chickpea cultivation in Northern India has been replaced in the aftermath of the green revolution with other rabi crops, especially irrigated wheat. This forced the shifting of chickpea cultivation from the traditional irrigated areas of Northern India to the non-traditional rainfed areas of Central India and Southern India. The crops in Central India and Southern India suffer from an array of abiotic stresses like heat and drought due to the climatic conditions of these states [10]. In India, drought has occurred at least once every 3 years during the last five decades; however, in Central India and Southern India, the incidence of drought is more recurrent [4,13]. The pre-flowering and early pod-filling periods of the chickpea crop are the most vulnerable to water stress [14].

Plants experience an array of morpho-physiological and biochemical changes in order to adapt to diverse abiotic challenges at the cellular level [15,16]. ROS (Reactive oxygen species), which are extremely poisonous and reactive molecules including superoxides, oxides, etc., that create oxidative stress in plants, are produced in excess. DNA, carbohydrates, lipids, and proteins are all severely damaged by ROS [17]. Remarkably, under severe environmental challenges, both enzymatic activities, including SOD, POD, CAT, and APX, along with non-enzymatic activities, viz., phenolic contents, ascorbic acid, carotenoids, glutathione, etc., are affected [18]. The overproduction of ROS and altered water interactions within the plant are the two main contributors to the cellular damage brought on by water shortage. Then, free ROS may target biological structures, causing DNA damage and oxidizing proteins and amino acids, inducing lipid peroxidation. Plants create ROS-detoxification mechanisms that work together to prevent ROS overproduction in order to prevent such harm [19]. Antioxidant defense systems maintain an equilibrium between the detoxification and production of ROS [20]. In chickpeas, abiotic stress tolerance mechanisms are correlated with the accumulation of osmolytes and antioxidants, which help in sustaining metabolism, protecting macromolecules, and retaining membrane integrity, leading to acclimation under drought stress [21,22]. Under drought stress, growth and photosynthesis are primarily affected; therefore, to understand the morpho-physiological and biochemical basis of yield variation and minimize yield loss under drought, it is imperative to evaluate growth parameters such as morpho-physiological and biochemical parameters along with yield-attributing traits [14,22,23].

Drought at the flowering stage in chickpeas is the main reason for a reduction in yield due to flower drop as well as limited pod formation. Apart from this, only a few studies have been conducted so far on desi chickpeas to understand the effects of drought during the flowering stage. Therefore, the current investigation was conducted with the following main objectives: (i) to assess the influence of drought pressure on physio-biochemical indices, (ii) to prioritize the physio-biochemical selection indices and yield-attributing traits for the acquisition of drought tolerance in chickpeas, and (iii) to screen flowering-stage drought-tolerant chickpea genotypes. Further, these prioritized selection indices would assist in the quick screening of the better-performing chickpea genotypes under the flowering stage drought stress. The identified best-performing genotypes may be further employed for sustainable production under climate change.

## 2. Results

### 2.1. Physiological Changes at Flowering Stage under Drought Stress Condition

**Figure 1:**
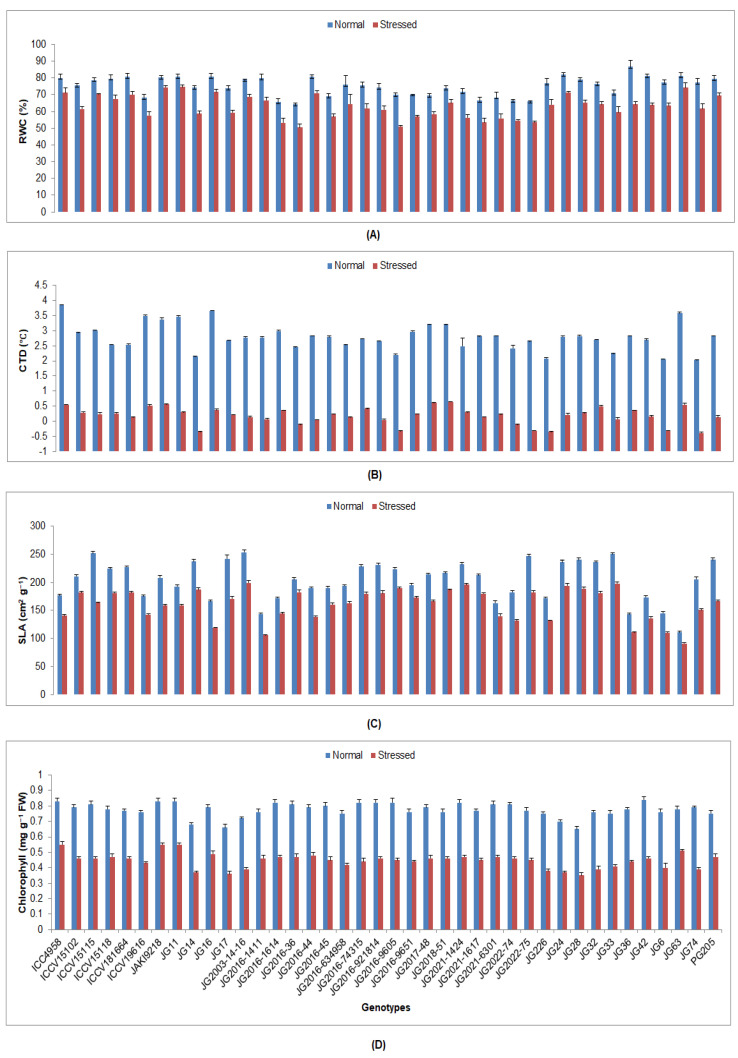
Consequence of drought stress at flowering stage on (**A**) RWC, (**B**) CTD, (**C**) SLA, and (**D**) Chl content of investigated genotypes, where RWC, CTD, SLA, and Chl designate relative water content, canopy temperature depression, specific leaf area, and chlorophyll, respectively.

**Figure 2:**
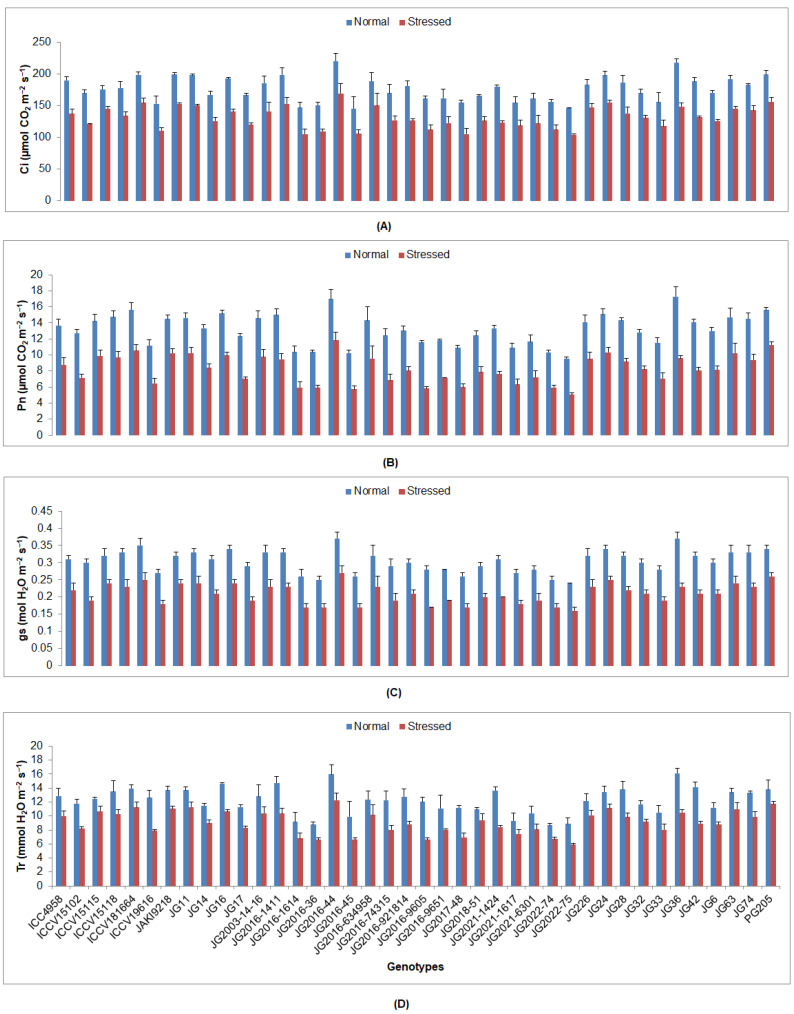
Effect of drought stress at flowering stage on (**A**) Ci, (**B**) Pn, (**C**) gs, and (**D**) Tr of experimented genotypes of chickpea, where Ci, Pn, gs, and Tr indicate the internal CO_2_ concentration, photosynthetic rate, stomatal conductance, and transpiration rate correspondingly.

All studied chickpea genotypes under drought stress at the flowering stage showed a significant decline in all investigated physiological traits compared to the plants grown under irrigated conditions (control) (Supplementary Tables S2 and S3). The maximum RWC was maintained by genotype JG11 (74.72%), whereas the minimum was perceived in JG2016-36 (50.47%) under stressed conditions (Figure 1). A higher CTD was depicted in genotype JG2018-51 (0.64 °C), whereas the lowest CTD was in JG74 (−0.39 °C). The maximum SLA was witnessed in genotype JG2003-14-16 (199.03 cm^2^g^−1^), while the lowest was in JG63 (90.93 cm^2^g^−1^). A higher Chl content was found in genotypes ICC4958 and JG11 (0.55 mg g^−1^ FW), whereas the lowest was seen in JG28 (0.35 mg g^−1^ FW). The highest Ci was sustained in genotype JG2016-44 (168.97 µmol CO_2_ m^−2^s^−1^), whereas the lowest was in JG2022-7 (104.01 µmol CO_2_ m^−2^s^−1^) (Figure 2). The maximum Pn was maintained by genotype JG2016-44 (11.82 µmol CO_2_ m^−2^s^−1^); however, the lowest was observed in JG2022-75 (5.06 µmol CO_2_ m^−2^s^−1^). A higher gs was portrayed in genotype JG2016-44 (0.27 mol H_2_O m^−2^s^−1^), whereas the lowest was presented by genotype JG2022-75 (0.16 mol H_2_O m^−2^s^−1^). A higher Tr was preserved in genotype JG2016-44 (12.26 mmol H_2_O m^−2^s^−1^), whilst the minimum was evident in genotype JG2022-75 (5.89 mmol H_2_O m^−2^s^−1^).

### 2.2. Biochemical Changes under Drought Stress Condition at Flowering Stage

**Figure 3:**
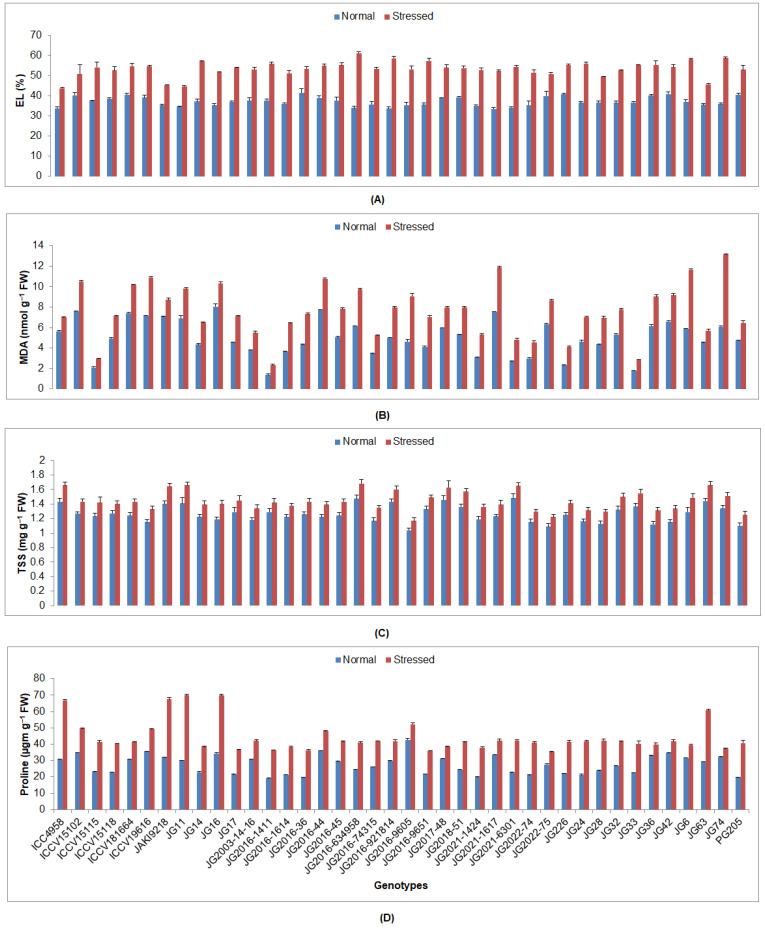
Influence of drought stress at flowering stage on (**A**) EL (%), (**B**) MDA, (**C**) TSS, and (**D**) proline content of investigated genotypes, where EL, MDA, and TSS indicate the electrolyte leakage, malondialdehyde, and total soluble sugar, correspondingly.

**Figure 4:**
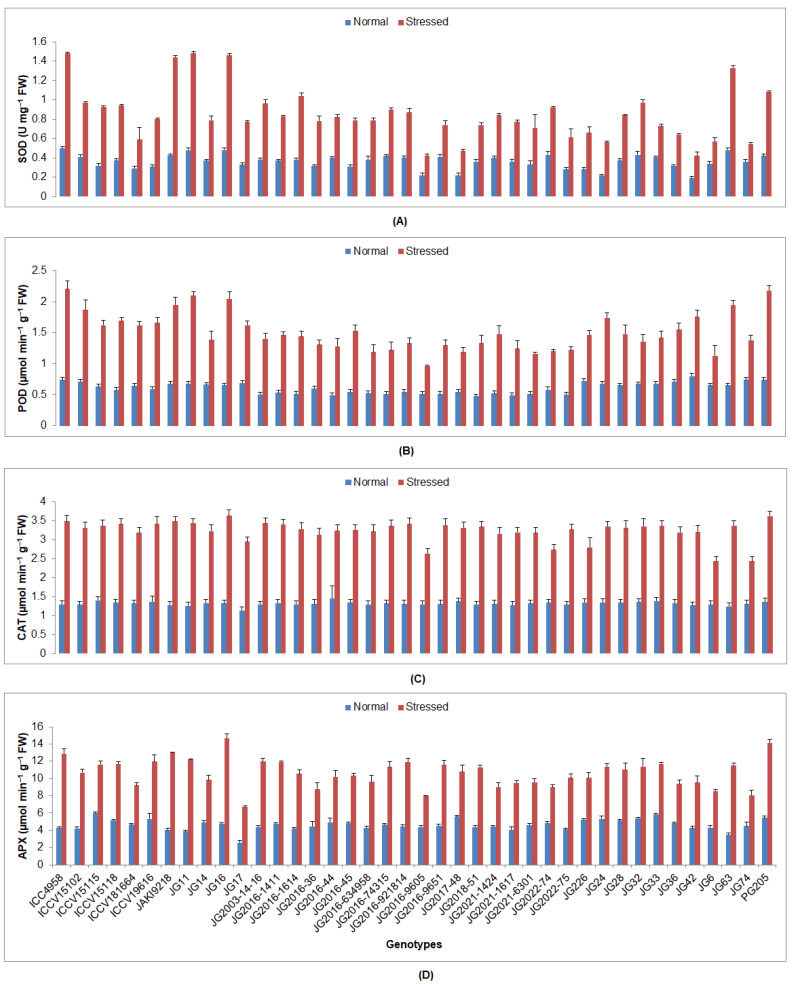
Consequence of drought stress at flowering stage on (**A**) SOD, (**B**) POD, (**C**) CAT, and (**D**) APX enzyme action of experimented genotypes, where SOD, POD, CAT, and APX indicate the superoxide dismutase, peroxidase, catalase, and ascorbate peroxidase, correspondingly.

All investigated biochemical traits were significantly increased under drought stress under normal irrigated conditions in all investigated genotypes (Supplementary Tables S4 and S5). The minimum EL content was observed in genotype ICC4958 (43.76%), while the maximum was detected in genotype JG2016-634958 (61.02%) under stressed conditions (Figure 3). The lowest MDA proportion was witnessed in JG2016-1411 genotype (2.32 nmol/g), whilst the maximum was presented by JG74 genotype (13.14 nmol/g). A higher sugar content was accumulated in genotype JG2016-634958 (1.68 mg/g FW), while the lowest was displayed by genotype JG2016-9605 (1.17 mg/g FW). The highest proline percentage was accredited to cultivar JG11 (69.91 µg/g FW), whereas the lowest was realized by genotype JG2022-75 (35.24 µg/g FW). The maximum SOD activity was witnessed by ICC4958 genotype (1.48 U/mg FW), whereas the lowest was documented in genotype JG42 (0.42 U/mg FW) (Figure 4). A higher POD activity was shown by genotype ICC4958 (2.21 µmol/min/g FW), whereas the lowest was demonstrated by genotype JG2016-9605 (µmol/min/g FW). The highest catalase activity was discerned by genotype JG16 (3.63 µmol/min/g FW), while the lowest was displayed by genotype JG74 (2.43 µmol/min/g FW). The highest APX activity was spotted in genotype JG16 (14.70 µmol/min/g FW), whereas the lowest was presented by genotype JG17 (6.69 µmol/min/g FW). 

### 2.3. Effect of Drought Stress at Flowering Stage on Yield and its Accrediting Traits

**Figure 5:**
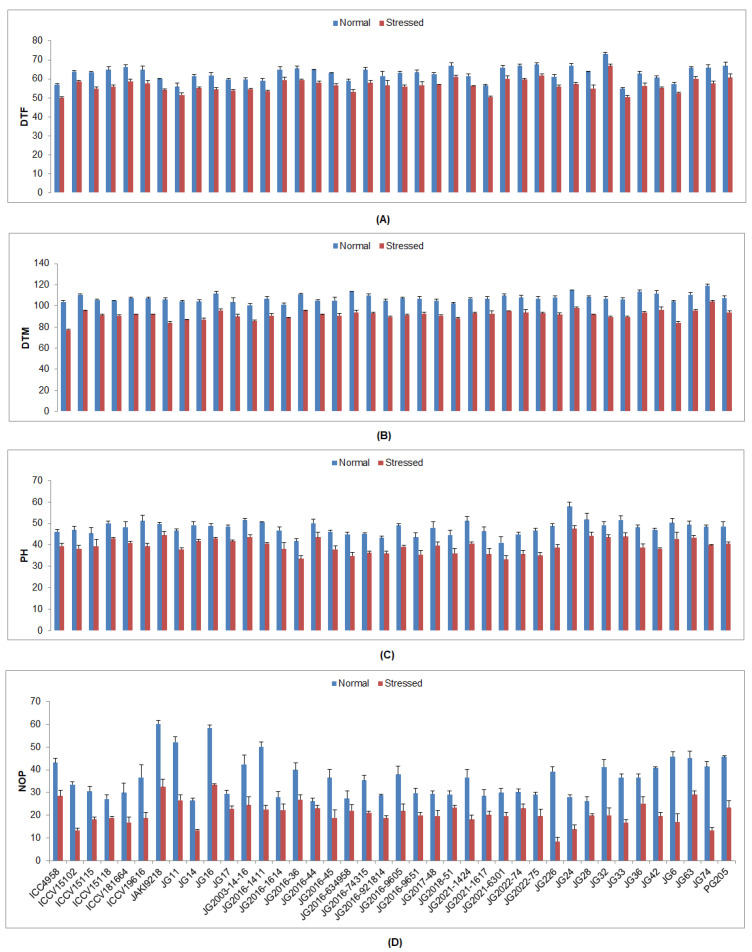
Influence of drought stress at flowering stage on (**A**) DTF, (**B**) DTM, (**C**) PH, and (**D**) NOP of investigated genotypes, where DTF, DTM, PH, and NOP designate days to 50% flowering, days to maturity, plant height, and numbers of pods, respectively.

**Figure 6:**
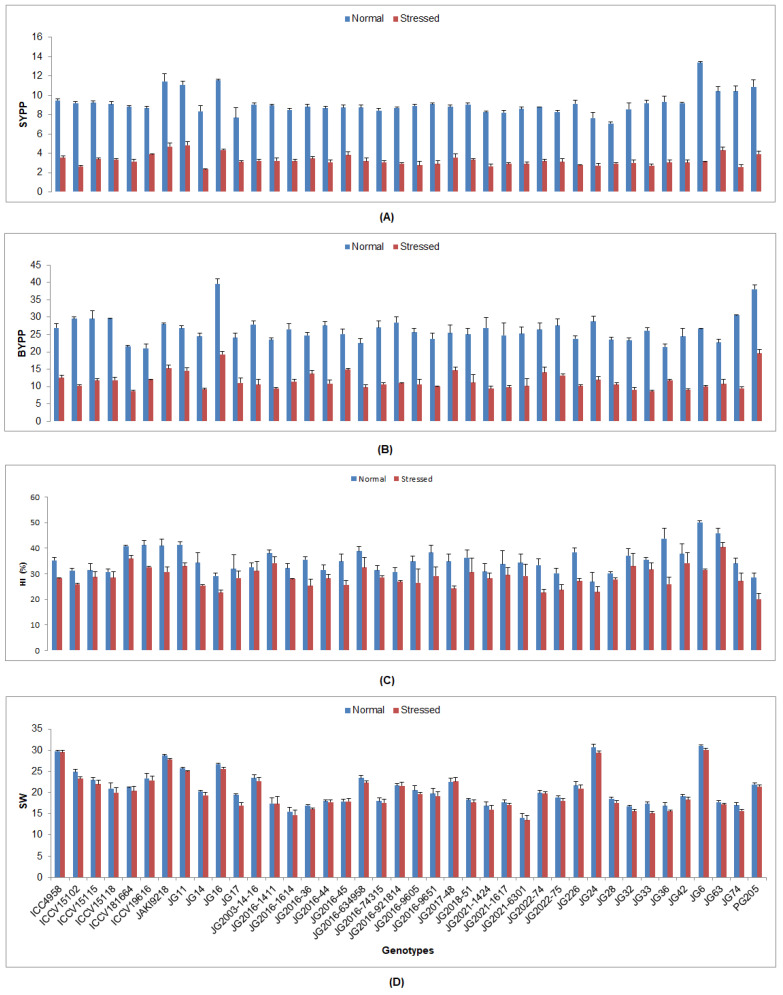
Influence of drought stress at flowering stage on (**A**) SYPP, (**B**) BYPP, (**C**) HI, and (**D**) SW of investigated genotypes, where SYPP, BYPP, HI, and SW designate seed yield per plant, biological yield per plant, harvest index, and 100-seed weight, respectively.

In all examined chickpea genotypes under drought stress, yield and responses to its determining traits were markedly reduced (Supplementary Tables S6 and S7). The lowest DTF was observed in genotype ICC4958 (49.95 DAS), whilst the highest was found in genotype JG32 (66.79 DAS) (Figure 5). The lowest DTM was depicted in genotype ICC4958 (77.17 DAS), while the most was presented by genotype JG74 (104.09 DAS). The lowest PH was documented in genotype JG2021-6301 (33.33 cm), whereas the maximum was shown by genotype JG24 (47.5 cm). The maximum NOP was counted in genotype JG16 (33.33), whereas the minimum was recorded in genotype JG226 (8.49). A higher SYPP was noticed in genotype JG11 (4.82 g), whilst the lowest was found in genotype JG14 (2.32 g) (Figure 6). A higher BYPP was witnessed in genotype PG205 (19.61 g), while the lowest was demonstrated by genotype JG33 (8.64 g). The maximum HI was demonstrated by genotype JG63 (40.42%), whereas the lowest was observed in genotype PG205 (20.08%). The highest SW was weighted in genotype JG6 (30.07 g), whilst the lowest was presented by genotype JG2021-6301 (13.50 g).

### 2.4. Prioritization of Traits Contributing to Chickpea Drought-Tolerance

**Figure 7:**
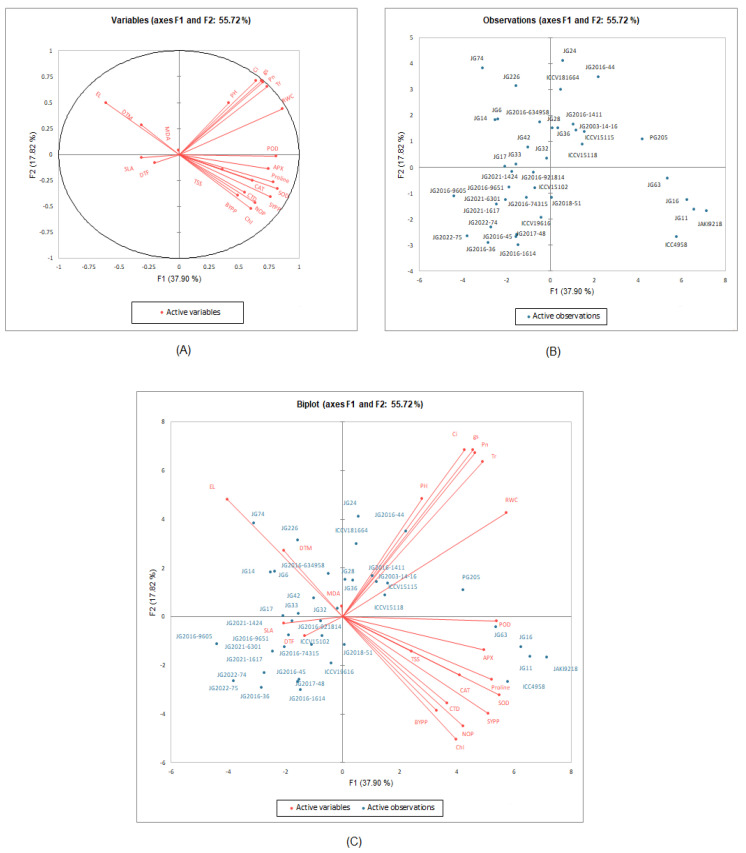
PCA biplots showing (**A**) relationship between the traits measured, (**B**) performance of chickpea genotypes, and (**C**) combined (**A**,**B**) under flowering stage drought-stressed condition. In the active variables, RWC, CTD, SLA, Chl, Ci, Pn, gs, Tr, EL, MDA, TSS, SOD, POD, CAT, APX, DTF, DTM, PH, NOP, SYPP, BYPP, HI, and SW indicate the relative water content, canopy temperature depression, specific leaf area, chlorophyll, internal CO_2_ concentration, photosynthesis rate, stomatal conductance, transpiration rate, electrolyte leakage, malondialdehyde, total soluble sugar, superoxide dismutase, peroxidase, catalase, ascorbate peroxidase, days to 50% flowering, days to maturity, plant height, number of pods, seed yield per plant, biological yield per plant, harvest index, and 100-seed weight, respectively.

The drought-linked traits were selected based on various physio-biochemical, yield, and its attributing trait responses in the present study. A PCA-based biplot analysis was performed for the selection of physio-biochemical, yield, and its accrediting traits contributing to drought tolerance in chickpea and selection made at the vegetative stage to search putative drought-tolerant chickpea genotype(s) (Figure 7). The angles of the trait vectors in PCA-biplot analysis indicate the correlation between variables. Angles between two trait vectors are correlated positively if they are less than 90°, negatively if they are greater than 90°, and independently if they are equal to 90°. Based on the vegetative stage drought stress response of the investigated chickpea genotypes, the PCA-biplot analysis clearly separated the features associated with the drought into strong positive correlations, positive correlations, independent correlations, and negative correlations. SY was strongly and positively correlated with CAT, SOD, APX, and POD activities along with proline and sugar accumulation, as well as NOP, CTD, and Chl contents. SY was also positively correlated with 100-SW, RWC, stomatal conductance, transpiration rate, and internal CO_2_ concentration. These accumulative, highly positive associated physio-biochemical, yield, and attributed parameters, which are more favored toward drought tolerance in chickpea genotypes, could be considered as the markers for terminal drought tolerance in chickpea. Based on physiological, biochemical, yield, and its corresponding phenotypic responses, the putative drought-tolerant genotypes were chosen for the vegetative stage. The PCA-based-biplot-selected genotypes were JG11, JAKI9218, JG16, JG63, PG205, and ICC4958 as vegetative-stage drought-tolerant chickpea genotypes based on the cumulative physio-biochemical, yield, and its attributing trait responses.

## 3. Discussion

Abiotic stresses cause morpho-physiological, biochemical, and molecular alterations that harmfully affect the development [24,25,26], efficiency, and, finally, yield of the crops [27,28]. Certain genotypes can withstand drought and continue to grow normally, but others cannot, which can have a significant impact on both growth and production. When sensitive cultivars are vulnerable to extreme drought circumstances, their nutritional qualities are also impacted, which could result in nutrient deficiencies. The identification and development of abiotic-stress-tolerant and climate-smart chickpea cultivars are necessary in the perspective of the occurrence of inconsistent patterns of rainfall, which is expected to increase soon due to climate change [21,29,30]. The chickpea has a narrow genetic variability, which largely affects chickpea improvement [31]. Low moisture stress affects the early vegetative growth stages of the chickpea plant [32], which ultimately affects yield [22]. Therefore, the physio-biochemical and agronomic performance of the selected chickpea genotypes was taken into consideration for the prioritization of drought-linked-selection indices and the identification of high-yielding chickpea genotypes with improved-vegetative-stage drought tolerance under drought stress.

The present findings agree with the earlier results that drought stress causes a substantial decrease in RWC, CTD, SLA, Ci, Pn, gs, Tr (mmol), Chl and protein content, DTF, DTM, NOP, SY, 100-SW, PY, and HI; as well as a substantial increase in hydrogen peroxide, electrolyte leakage, and MDA content [31,33]; and an accumulation of osmolytes, i.e., sugar and proline content. The activity of antioxidant enzymes including SOD, POD, CAT, and APX in all studied genotypes [14] was also reduced. Similar results have been found in a recent study conducted by Alsamadany et al. [34] in tomato plants. The decline in the activities of antioxidant enzymes under drought conditions may be due to the production of ROS, which may be a causal factor for the reduction in their activities. The decline in plant growth may be the result of a negative correlation between plant response and the availability of soil moisture content. This might be explained by a decrease in cell growth and increased leaf senescence in plants’ underwater stress [35]. A positive correlation between high early vigor and plant yield was already established [36,37]. According to earlier studies, irrigation application had beneficial impacts on phosphate solubilization, microorganism activity, crop growth, and development in chickpea cultivars [38,39]. Another report elaborated that irrigation supplementation significantly enhanced the numbers of nodules and seed yield in chickpea [22,40]. In maize, sorghum, and soybean, plant height, leaf area, dry matter, plant growth, and, ultimately, yield were diminished in susceptible genotypes under low soil moisture content [41,42]. These parameters were also studied in chickpea crop and similar findings were published by various researchers. According to their reports, the lack of water caused all generative portions of the plant to shrink, which had a negative impact on the crop’s yield more so at the flowering stage than the vegetative stage. This is evident for the number of flowers/plant parameter, which has shortened the flowering time and resulted in floral abortion due to drought during flower induction [21,23,28,43]. These reports demonstrated the reasons for the ultimate yield reduction in chickpea under water stress conditions. Photosynthesis plays a crucial role in determining growth and development in plants [15]. Many studies showed that abiotic stress led to a decrease in photosynthesis rate, which may be assessed by examining photosynthetic pigments. Under stress conditions, chlorophyll ‘a’ and chlorophyll ‘b’ were lesser reduced in tolerant genotypes. A prior study with similar findings also showed that chickpea heat tolerance genotypes had higher chlorophyll levels than sensitive genotypes [44]. Our findings are also in agreement with the results of Sree et al. [33] where chlorophyll content decreased in the chickpea cultivars under water stress conditions. The contents of photosynthetic pigments, *viz*., chlorophyll ‘a’ and ‘b’, are directly related to water stress tolerance. The reduction in chlorophyll contents may be because of the disturbance in biosynthesis or their breakdown under water stress. A decline in chlorophyll content under water-stressed plants of different crops was also reported by Sree et al. [33].

A significant reduction was observed in protein content under water stress conditions in the present investigation. It was previously observed that the low water status in plants resulted in a considerable decrease in protein production, which may have been caused by an array of factors [45]. Protein molecules play a vital role in proper functions of cells [43]. Since proteins directly influence the development of novel phenotypes by modifying physiological features in response to environmental changes, their role is essential in the stress responses of plants [46]. Abiotic stressors have been found to cause the misfolding of freshly synthesized proteins and the denaturation of already-existing proteins [27,47]. This reduction in protein content ultimately leads to a reduction in plant growth and crop yield in sensitive chickpea genotypes [48]. Under drought stress, protease and other catabolic enzymes may become more active, leading to increased protein breakdown. Alternatively, reactive oxygen species may fragment proteins, causing a decrease in protein content. In drought-stressed plants, a decrease in protein content has frequently been distinguished as a sign of oxidative stress [49]. The tolerant genotypes that show less reduction in protein content may have better adaptability under water stress conditions [50]. Soluble proteins may repair drought-induced membrane injuries under stress in tolerant genotypes [51]. The results of the current investigation are consistent with those of the prior investigation in lucerne (*Medicago sativa*), in which it was found that genotypes with the highest accumulations of soluble protein were thermos-tolerant [52]. In this experiment, we saw a decline in the relative water content in some genotypes under water stress circumstances. It is already reported that water stress negatively affects relative water content in chickpea [22]. Under low moisture content in the soil, transpiration rate and leaf RWC were decreased, which consequently increased the leaf canopy temperature [53]. When there is water shortage, a plant performs better if its relative water content is high. Most researchers have found that leaves respond to drought stress by losing relative water content and water potential. It is believed that cultivars displaying a higher relative water content under drought stress are more resilient and yield more than others [32,54].

Crops have become more prone to oxidative damage because of unpredictable climate change through excessive production of toxic ROS such as H_2_O_2_, superoxide, and hydroxyl radicals [55]. According to earlier studies on chickpea, increased oxidative stress led to an increase in the oxidant status of sensitive chickpea genotypes under low-moisture-stress conditions [22,44]. Malondialdehyde (MDA), a molecule produced by membrane lipids in reaction to reactive oxygen species (ROS), can be used as a drought indicator to gauge the extent of plasma membrane damage and the ability of plants to tolerate drought stress [56]. Lipid peroxidation and MDA concentration were positively correlated, and the latter can degrade the strength of the cell wall [21,57]. The findings of our investigation are consistent with a prior study, which found that the tolerant chickpea genotypes accumulated lower levels of MDA than sensitive genotypes [58]. In our study, the effect of drought stress was demonstrated least in drought-tolerant genotypes in comparison to other genotypes, which might be due to the accumulation of osmolytes and the enhanced activities of antioxidant enzymes. Chickpea-stress-tolerant genotypes accumulate more osmolytes (osmotically active compounds with low molecular weight) such as proline and glycine betaine and exhibit a reduction in the level of glutathione [59,60]. To detoxify the effects of ROS generated under abiotic stresses, specialized antioxidant enzymes, viz., SOD, CAT, POD, and APX, become triggered and act as a first line of defense [61]. In the chickpea genotypes, Rasool et al. [62] showed a considerable increase in SOD activity, indicating that SOD may work as an ROS scavenger by converting O_2_^−^ to H_2_O_2_. Similar results were also obtained by Nazar et al. [63] in *Vigna radiata* where SOD activity was increased in response to stress. SOD converts superoxide radicals into H_2_O_2_, which is further reduced to water by POD and CAT [64]. When compared to irrigated settings, genotypes that are resilient to drought stress showed increased antioxidant enzyme activities. It has been previously established that enhanced antioxidant enzyme activity is highly linked with chickpea drought resistance [65,66]. In Osage orange (*Maclurapomifera*), SOD activity increased in association with a drop in moisture content [67]. A significant increase in POD was also found under the combined (drought plus heat) stress in D-09027 and CH24/07 genotypes in comparison to the control [22].

Drought stress reduces the yields of plants, especially grain legumes [68]. The main cause of this decrease in yield of grain legumes during drought circumstances is the reduction in the numbers of pods per plant. Under stressed conditions, the maximum plant yield per plant was observed in genotypes JG11, JAKI9218, G16, JG63, and ICC4958 with lesser reductions in RWC, photosynthetic rate, number of secondary branches per plant, and biomass; higher accumulations of osmolytes, viz., sugar and proline; and higher activities of antioxidant enzymes including SOD, POD, CAT, and APX. Our findings are in agreement with various previous studies. The seed yield in tolerant genotypes of chickpea was observed to range from 2.97 to 5.73 percent and, in susceptible genotypes, ranged from 9.2 to 24.66 percent [69]. A significant reduction in yield traits leads to a decline in plant yield under water stress conditions. The genotypes that were subjected to drought stress reached maturity earlier and had shorter life cycles, fewer pods, and smaller seeds. The water stress condition reduced pre-flowering and the number of days before flowering, but flowering-stage drought stress reduced the time until seed germination [33].

By demonstrating the relationships between the morpho-physiological and biochemical parameters and the distribution patterns of chickpea genotypes under drought-stressed conditions, PCA-biplots demonstrated an excellent contribution to the performance of chickpea genotypes under drought stress. The most effective multivariate methodology for assessing the relationship between genotypic performance and characteristics is PCA-biplot [14]. Numerous researchers are making full use of it to analyze the characteristics’ association in various crop plants [70]. The strong positive relationship of SYPP was revealed through biplot analysis based on principal component and correlation analysis with RWC, Pn, gs, Ci, Chl content, TSS and proline content, antioxidant enzyme activities, NOP, 100-SW, and BYPP, suggesting their greater utilization in selecting putative drought-tolerant genotypes [71]. A new insight into the mechanisms behind drought tolerance and the responses of plants to drought stress was offered through PCA-biplots. Genotypes, viz., ICC4958, JAKI9218, JG11, JG16, and JG63, were detected as the most drought-tolerant genotypes at both early and terminal drought stress stages. Our results confirm those of Sachdeva et al. [23], who, through principal component analysis based on biplot and correlation analysis, found strong positive associations with RWC, chlorophyll index (CI), membrane stability index (MSI), secondary branches (SBs), and yield traits and negative associations with drought susceptibility index (DSI), 100-SW, and days to maturity under drought stress. The most drought-tolerant genotypes identified were ICC4958, Pusa1103, BGD72, CSG8962, ICCV97309, ICCV10, ICCV03311, ICCV05308, ICCV3403, and ICCV10313 based on PCA-biplot analysis. These genotypes had lower values of DSI and DTM and high RWC and MSI values under drought stress conditions under both vegetative and reproductive stages. In a similar manner, Shah et al. [70] used biplot analysis to identify superior chickpea genotypes under drought stress and concluded that the genotypes, viz., D0091-10, D0085-10, K010-10, K005-10, 08AG016, D0078-10, 08AG004, 09AG002, D0080-10, K002-10, and D0099-10, were superior in terms of yield and physio-biochemical performance. Furthermore, genotype-by-trait (GT) biplots were created for the more accurate identification of genotypes with the highest value for multiple characteristics in chickpea for all genotypes under stress situations [22].

## 4. Materials and Methods

This study was conducted at the Biotechnology Centre of Jawaharlal Nehru Krishi Vishwa Vidyalaya, JNKVV, Jabalpur, Madhya Pradesh, India in a completely randomized design (CRD) with three replications to examine the effects of normal irrigated and drought-stressed treatments on the physiology, biochemistry, yield, and its attributing traits of desi chickpea at reproductive stage during Rabi 2020–2021 and 2021–2022. The All India Coordinated Research Project (AICRP) on Chickpea, Lead Centre, Department of Plant Breeding and Genetics, JNKVV, Jabalpur, Madhya Pradesh, India, provided forty chickpea genotypes, including drought-tolerant check, released varieties, and advanced breeding lines that were used as experimental materials (Supplementary Table S1). Seeds were surface-sterilized using Bavistin @ 2.0 g per kg seed, Chlorpyriphos 20EC @ 10 mL per kg seed, and Rhizobium @ 5 g per kg seed. Five seeds per pot were sown in 45 × 20 × 20 (L × W × H) cm pots containing 10 kg of homogenized sandy clay loam soil (mixed with vermicompost and cow dung). First thinning was performed after 14 days of seed germination to sustain 4 seedlings per pot, while second thinning was carried out after a week of first thinning to retain three uniform seedlings per pot for subsequent studies. Each pot was irrigated regularly with tap water to 75–80% field capacity (FC) until commencement of drought treatments. Drought stress was imposed 40 days after sowing (DAS) by withholding irrigation to stressed pots (until soil moisture attained 35–40% FC of soil), while regular irrigation to normal irrigated pots was continued. Data on the following physio-biochemical and yield-attributing traits were recorded.

### 4.1. Physiological Traits

From each treatment, three plants were randomly selected for the recording of different physiological traits.

#### 4.1.1. Relative Water Content (RWC)

To measure RWC, 400 mg of fresh leaf samples were transferred to Petri plates containing distilled water at room temperature (RT). The turgid weight was observed after an incubation of leaf samples for 4 h. For recording dry weight, oven drying of the leaf samples was performed at 60 °C for 72 h. RWC was calculated utilizing the following formula [72].

RWC = [(Fresh weight − Dry weight)/(Turgid weight − Dry weight)] × 100

#### 4.1.2. Canopy Temperature Depression (CTD)

Leaf canopy temperature (Tc) and air temperature (Ta) were measured using spectrum thermometer, and CTD was calculated according to formula proposed by Ramamoorthy et al. [73].

CTD = (Ta) − (Tc)

#### 4.1.3. Gas Exchange Parameters

The leaf gas exchange parameters were assayed in the fully expanded upper-third leaf of the normal and stressed plants. The net photosynthesis rate (Pn; μmol CO_2_ m^−2^s^−1^), stomatal conductance (gs; mol H_2_O m^−2^s^−1^), transpiration rate (Tr; mmol H_2_O m^−2^s^−1^), and internal CO_2_ concentration (Ci; μmol CO_2_ m^−2^s^−1^) were analyzed using a portable infra-red gas analyzer (IRGA) LiCor-6400 (LiCor Instruments, Lincoln, NE, USA).

### 4.2. Biochemical Traits

From each treatment, three plants were randomly selected for recording biochemical traits.

#### 4.2.1. Chlorophyll Content

A hundred milligrams of leaf samples were homogenized in 1 mL of 80% (*v*/*v*) acetone and incubated on a shaker for an overnight period at RT (tubes were covered with foil to keep away from light). The homogenate was centrifuged for ten minutes at 13,000 rpm. The absorbance was recorded at 645 and 663 nm wavelengths using UV/Vis spectrophotometer (Jasco, V-550, Oklahoma City, OK, USA) for measuring the amount of photosynthetic pigment according to Arnon’s [74] equation.

#### 4.2.2. Determination of Oxidative Stress by Measuring Electrolyte Leakage (EL) and Lipid Peroxidation (Malondialdehyde Content)

By using distilled water, five fresh leaves were carefully washed before being placed in test tubes with 10 mL of water. The test tubes were maintained at two different temperature regimes, i.e., 45 °C and 100 °C for 30 min and 10 min, respectively, in a water bath as per the method suggested by Sachdeva et al. (2022). Then, electrical conductivities (L_1_) and (L_2_) were recorded using an AL20ConAQUALYTIC (Dortmund, Germany), Portable Conductivity Meter. The EL was calculated using the formula given by Lutts et al. [75].

EL (%) = (C_1_/C_2_) × 100

According to the protocol suggested by Naserwafaei et al. [76], malondialdehyde (MDA), a byproduct of unsaturated fatty acid peroxidation content, was used to measure lipid peroxidation. One milliliter of 20% *w*/*v* trichloroacetic acid was used to homogenize 100 mg of leaf samples before they were centrifuged at 15,000× *g* for 10 min at 4 °C. The TCA (20% *w*/*v*) was combined with an equivalent volume of supernatant and 5% *w*/*v* TBA. The combination was heated at 96 °C for 30 min before spending 5 min in an ice bath. Both the primary absorbance at 532 nm and the correction absorbance at 600 nm for non-specific turbidity were measured. MDA concentration was calculated using the following formula.

MDA nmol g^−1^ FW = ((A532 − A600) × V × 1000/E) × W

where A600 is the absorbance at 600 nm, A532 is the absorbance at 532 nm, W is the fresh weight of the leaf, and E is the specific extinction coefficient (155 mM cm^−1^).

#### 4.2.3. Determination of Osmolytes Content by Measuring Free Proline and TSS Content

For determining total soluble sugar content, 0.1 mL of the alcoholic extract of leaf tissue was prepared by homogenizing 0.1 g of leaf samples in 0.5 mL of absolute ethanol, vortexed for 1 min and centrifuged at 13,000 rpm for 10 min, and subsequently treated with 3.0 mL of freshly made anthrone reagent prepared by dissolving 150 mg of anthrone in 100 mL of 72% H_2_SO_4_. Glass test tubes were incubated at 100 °C for 10 min and cooled for measuring OD at 620 nm using the UV–Vis spectrophotometer (Jasco, V-550). The calibration curve prepared with known concentrations of glucose (Himedia, Mumbai, India) was employed for calculation of TSS quantity as suggested by Shukla et al. [77].

Free proline contents of leaf were determined using ninhydrin according to the method suggested by Mishra et al. [78]. One hundred milligrams of leaves were homogenized in 1.2 mL of 3% aqueous sulfosalicylic acid and centrifuged at 13,000 rpm for 10 min. A 0.5 mL supernatant was made up to 1.0 mL by supplementing 0.5 mL of distilled water and reacted with 1.0 mL of 2% ninhydrin to incubate at 90 °C for 1 h. Two milliliters of toluene was added to the samples after cooling the reaction mixture in an ice bath and vortexed for 2 min. The supernatant was taken for recording the absorbance at 520 nm using a UV–Vis spectrophotometer (Jasco, V-550) and free proline content was calculated using the standard curve prepared with known concentrations of L-proline (Himedia, Mumbai, India).

#### 4.2.4. Antioxidant Enzyme Activities

One gram of fresh leaf samples were ground in liquid nitrogen and transferred immediately in a 10 mL chilled enzyme extraction buffer comparing 50 mM potassium phosphate buffer, pH 7.5, 0.5 mM EDTA, and 1% polyvinylpyrrolidone in the case of SOD, POD, and CAT, while 1 mM ascorbic acid was also added in the case of APX and centrifuged at 13,000 rpm for 20 min at 4 °C as per the method described by Sharma et al. [79]. Brie was passed through 4 layers of cheesecloth and the supernatant was employed as crude enzyme extract to determine the enzymatic activities.

SOD activity was estimated according to the method proposed by Dhindsa et al. [80] by recording the decline in observance of formazone by the enzyme made by the reaction of superoxide radical and nitro-blue tetrazolium (NBT) dye. The reaction was started by adding 0.1 mL of 2 μM riboflavin to 3.0 mL of the reaction mixture (13.33 mM methionine, 75 µM NBT, 0.1 mM EDTA, 50 mM phosphate buffer, pH 7.8, 50 mM sodium carbonate, 0.1 mL of enzyme, and 0.9 mL of water) and putting the glass tubes under a fluorescent chamber made by using two 15 W fluorescent lamps for 15 min. A complete reaction mixture without the enzyme that produced the most color was used as the control, and a complete reaction mixture that had not been exposed to radiation was used as a blank. The reaction was stopped by putting the tubes in the dark, and the absorbance at 560 nm was recorded. The amount of enzyme, measured as one unit of enzyme activity, caused the absorbance to decrease by 50% when compared to the control.

POD activity was assayed based on the oxidation of guaiacol to tetra-guaiacol [81]. Fifty micromoles of phosphate buffer, 16 mM guaiacol, 2 mM H_2_O_2_, and 0.1 mL of enzyme extract were added and the volume was made up to 3.0 mL of reaction mixture. The pH of the mixture was maintained at 6.1. An increase in absorbance was recorded at 470 nm using the UV–Vis spectrophotometer (Jasco, V-550) to calculate the enzyme activity as per extinction coefficient of tetra-guaiacol ∈ = 26.6 mM^−1^ cm^−1^.

CAT activity was assayed from the decrease in the absorbance over a time period as suggested by Aebi [82]. To start the reaction, 0.05 mL of crude enzyme extract was added to 3.0 mL of reaction mixture that consisted of 50 mM potassium phosphate buffer, 12.5 mM hydrogen peroxide, and 0.5 mL of water, and the decrease in absorbance was recorded at 240 nm for 1 min using the UV–Vis spectrophotometer (Jasco, V-550). Enzyme activity was calculated as the amount of H_2_O_2_ decomposed per minute.

Ascorbic acid breakdown was used to measure APX activity from the decrease in absorbance. To start the reaction, 0.2 mL of hydrogen peroxide was added to the 3.0 mL reaction mixture containing 50 mM potassium phosphate buffer, pH 7.0, 0.5 mM ascorbic acid, 0.1 mM EDTA, 0.1 mM H_2_O_2_, 0.1 mL of enzyme, and 0.7 mL of water. A decrease in absorbency was recorded for a period of 30 sec at 290 nm using the UV–Vis spectrophotometer (Jasco V-550) to calculate the enzymatic activity as per extinction coefficient of ascorbate ∈ = 2.8 mM^−1^cm^−1^.

### 4.3. Phenological Traits

From each treatment, three plants were randomly selected for recording phenological traits. Days to 50% flowering and days to maturity were measured as the number of plants in a replication that had at least one opened flower and the number of plants whose pods turned brownish yellow from the DAS, respectively. The number of first-order and second-order branches that emerged from the main shoot at maturity was counted to determine the equivalent amounts of primary and secondary branches per plant, respectively.

### 4.4. Yield and Its Attributing Traits

Three plants were randomly chosen from each treatment at harvest to record yield and its accrediting characteristics. Number of pods per plant (NOPPP) is the total number of seed-filled pods on a plant, while biological yield per plant (BYPP) is the whole weight of the plant, including the pods. A plant’s harvested seeds were weighed to determine the seed yield per plant (SYPP), and the weight of 100 seeds from each treatment’s seed lot was recorded as the treatment’s 100-seed weight (SW). The ratio of the biological yield per plant to the seed yield per plant was multiplied by 100 to obtain the harvest index (%).

### 4.5. Statistical Analysis

Three replications of a completely randomized design (CRD) were employed to screen different chickpea genotypes. In Rabi 2020–2021 and 2021–2022, several morpho-phenological, physio-biochemical, and yield-related parameters associated with the drought were recorded. All 40 genotypes of chickpeas were polled for both water conditions during both seasons. The significance was established through analysis of variance (ANOVA) and Duncan Multiple Range Test (DMRT) at *p* < 0.05 by using STAR V2.0.1 and SPSS V20 software, respectively. In order to prioritize the most trustworthy selection indices in drought-stressed conditions, PCA and PCA-based biplots were also created using the XLSTAT premium program.

## 5. Conclusions

It is crucial to find new genetic resources that can withstand drought-stress situations. Early growth stage characteristics did not guarantee good yielding or drought tolerance at the end of the life cycle. The primary focus of this study was on locating appropriate physiological and biochemical indicators that can be used to discriminate between the tolerant and susceptible genotypes. The present investigation revealed that RWC, CTD, and CCI; gas exchange parameters including Pn, gs, Tr, and Ci; along with biochemical parameters, viz., proline, protein, total soluble sugar, and antioxidant enzyme activities could distinguish between tolerant and sensitive genotypes. The study of morpho-physiological, biochemical, and yield-associated traits demonstrated that genotypes, viz., ICC4958, JAKI9218, JG11, JG16, JG63, and PG205, could be considered drought-tolerant. The identified genotypes of chickpea could be used in future crop development initiatives.

## Data Availability

All necessary data supporting the conclusions of this article will be available from the authors without undue reservation.

## Supplementary Materials

The following supporting information can be downloaded at https://www.mdpi.com/article/10.3390/plants12183175/s1, Table S1: Details of 40 chickpea genotypes used for present study; Table S2: Pooled physiological responses of various chickpea genotypes under normal irrigated condition; Table S3: Pooled physiological responses of various chickpea genotypes under drought stress at vegetative stage; Table S4: Pooled biochemical responses of various chickpea genotypes under normal irrigated condition; Table S5: Pooled biochemical responses of various chickpea genotypes under drought stressat vegetative stage; Table S6: Pooled yield and its attributing trait responses of various chickpea genotypes under normal irrigated condition; Table S7: Pooled yield and its attributing trait responses of various chickpea genotypes under drought stressat vegetative stage.
