# Supplementary material for: Prioritization of Physio-Biochemical Selection Indices and Yield-Attributing Traits toward the Acquisition of Drought Tolerance in Chickpea (Cicer arietinum L.)
Source: Plants (Basel). 2023 Sep 5;12(18):3175. doi: 10.3390/plants12183175 (PMC10534892; doi:10.3390/plants12183175)
Supplement: Supplementary file 1 [file plants-12-03175-s001.zip › plants-2585535-supplementary.pdf]

Supplementary Table S1 Details of 40 chickpea genotypes used for present study

S.No.	Genotype	Pedigree	Origin	Status
1	ICC4958	JGC 4958	JNKVV, Jabalpur	Genetic stock (1973)
2	JAKI9218	(ICCC 37 x GW5/7) x ICCV 107	JNKVV, Jabalpur	Released variety (2006)
3	JG11	(Phule G-5 x Narsinghpur bold) x ICCC 37	JNKVV, Jabalpur	Released variety (1999)
4	JG16	ICCC 44 × ICCV 10	JNKVV, Jabalpur	Released variety (2001)
5	JG63	Single Plant selection from JG 62	JNKVV, Jabalpur	Released variety (2004)
6	JG74	A composite from genetic stock	JNKVV, Jabalpur	Released variety (1983)
7	JG6	(ICCV10XK850) x (H208XRS11)	JNKVV, Jabalpur	Released variety (2008)
8	JG14	(GW5/7XP326)XICCL83149	JNKVV, Jabalpur	Released variety (2009)
9	JG17	BDNG 9-3 x Narshingpur Bold	JNKVV, Jabalpur	Released variety
10	JG24	(JG 74 x ICC 4958)-21	JNKVV, Jabalpur	Released variety (2019)
11	JG28	[(JM – 1 X IPC 9239)X JG 7] – 14-11	JNKVV, Jabalpur	Released variety
12	JG32	[(JM – 1 x IPC 4958) x JG 315] – 2	JNKVV, Jabalpur	Released variety
13	JG33	[(JM – 1 x IPC 9239) x JG 322] – 30-3	JNKVV, Jabalpur	Released variety
14	JG36	JG 12 x JG 16	JNKVV, Jabalpur	Released variety (2017)
15	JG42	[(JM 1x IPC 9239) JG7] 14-11-2011-42	JNKVV, Jabalpur	Released variety
16	JG226	JG 74 x JG315	JNKVV, Jabalpur	Released variety (2007)
17	PG205	JG 315 x ICC 96029	JNKVV, Jabalpur	Released variety
18	ICCV15102	ICCV03112 x ICCV10	ICRISAT, Patancheru	Advanced breeding line
19	ICCV15115	ICCV10 x ICCV 96970	ICRISAT, Patancheru	Advanced breeding line
20	ICCV15118	ICCV 05530 x ICCV 88510	ICRISAT, Patancheru	Advanced breeding line
21	ICCV19616	JAKI 9218/ICCV 05103	ICRISAT, Patancheru	Advanced breeding line
22	ICCV181664	ICC 4958 TM/JG 130	ICRISAT, Patancheru	Advanced breeding line
23	JG2003-14-16	[(JM1 x ICC4929) x ICC4958]-2-14-16	JNKVV, Jabalpur	Advanced breeding line
24	JG2016-44	(ICC 96029 x ICC11551) 44	JNKVV, Jabalpur	Advanced breeding line
25	JG2016-45	(JG 74 x ICC11551) 45	JNKVV, Jabalpur	Advanced breeding line
26	JG2016-1411	JG 14 x JG 11	JNKVV, Jabalpur	Advanced breeding line
27	JG2016-1614	JG 16 x JG 14	JNKVV, Jabalpur	Advanced breeding line
28	JG2016-9605	JG 74 x ICC 96029	JNKVV, Jabalpur	Advanced breeding line
29	JG2016-9651	JG 130 x ICC 96029	JNKVV, Jabalpur	Advanced breeding line
30	JG2016-74315	[{(JG 74 x WR 315) x JG 74} -2010 -1- 3- 5- 11-15-10-2]	JNKVV, Jabalpur	Advanced breeding line
31	JG2016-634958	JG 63 x ICC 4958	JNKVV, Jabalpur	Advanced breeding line
32	JG2016-921814	JAKI 9218 x JG 14	JNKVV, Jabalpur	Advanced breeding line
33	JG2017-48	(JG 315 x ICC 96029)48	JNKVV, Jabalpur	Advanced breeding line
34	JG2018-51	JG63 x ICC1205	JNKVV, Jabalpur	Advanced breeding line

35	JG2022-74	JG12XJG14	JNKVV, Jabalpur	Advanced breeding line
36	JG2016-36	JG12XJG16	JNKVV, Jabalpur	Advanced breeding line
37	JG2022-75	JG12XICC4958	JNKVV, Jabalpur	Advanced breeding line
38	JG2021-6301	JG12XICCV06301	JNKVV, Jabalpur	Advanced breeding line
39	JG2021-1424	JG14XJG24	JNKVV, Jabalpur	Advanced breeding line
40	JG2021-1617	JG16 X JG17	JNKVV, Jabalpur	Advanced breeding line

Supplementary Table S2 Pooled physiological responses of various chickpea genotypes under normal irrigated condition

**Genotypes**	**RWC**	**CTD**	**SLA**	**Ci**	**Photo**	**Cond**	**Trmmol**
ICC4958	80.05±2.29^{klmno}	3.85±0.02^{r}	176.84±1.43^{d}	189.99±5.71^{ijkl}	13.67±0.83^{klmnop}	0.31±0.01^{jklm}	12.87±1.15^{ghijklmn}
ICCV15102	75.71±0.92^{ghi}	2.93±0.02^{m}	210.78±2.93^{hi}	169.97±4.63^{defgh}	12.75±0.43^{ghijk}	0.3±0.01^{ghijk}	11.78±0.6^{defghij}
ICCV15115	78.81±1.13^{hijklmno}	3.01±0.01^{m}	251.24±3^{r}	175.47±5.68^{efghi}	14.24±0.79^{mnopqr}	0.32±0.02^{lmno}	12.46±0.21^{ghijkl}
ICCV15118	79.63±2.12^{jklmno}	2.54±0.01^{f}	224.34±2.42^{k}	177.59±9.97^{fghij}	14.73±0.8^{pqrs}	0.33±0.01^{lmno}	13.52±1.53^{ijklmn}
ICCV181664	80.85±2.03^{mno}	2.53±0.04^{f}	227.47±1.73^{kl}	198.46±4.76^{kl}	15.67±0.83^{s}	0.35±0.02^{o}	13.94±0.49^{klmn}
ICCV19616	68.33±2.02^{bcde}	3.49±0.04^{p}	175.46±1.67^{d}	152.92±11.77^{abc}	11.18±0.69^{bcdef}	0.27±0.01^{bcdef}	12.6±1.12^{ghijkl}
JAKI9218	80.43±1.06^{lmno}	3.38±0.04^{o}	208.06±3.9^{gh}	198.84±2.99^{kl}	14.49±0.53^{opqrs}	0.32±0.03^{lmno}	13.72±0.56^{jklmn}
JG11	80.75±1.43^{mno}	3.47±0.03^{p}	192.54±3.4^{f}	197.66±2.32^{kl}	14.6±0.68^{opqrs}	0.33±0.01^{lmno}	13.69±0.49^{jklmn}
JG14	74.11±1.17^{fg}	2.14±0.01^{bc}	237.03±4.05^{no}	166.89±5.42^{cdefg}	13.32±0.44^{jklmno}	0.31±0.04^{ijkl}	11.47±0.38^{defgh}
JG16	80.9±1.92^{mno}	3.66±0.02^{q}	166.3±1.73^{c}	192.59±2.15^{jkl}	15.19±0.43^{qrs}	0.34±0.01^{no}	14.63±0.16^{mno}
JG17	73.69±1.49^{fg}	2.68±0.01^{gh}	241.41±6.44^{op}	166.47±2.68^{cdef}	12.33±0.34^{fghij}	0.29±0.01^{efghi}	11.28±0.37^{defg}
JG2003-14-16	78.85±0.33^{hijklmno}	2.78±0.02^{ijkl}	252.96±4.34^{r}	185.33±11.76^{hijkl}	14.56±0.96^{opqrs}	0.33±0.02^{lmno}	12.82±1.65^{ghijklmn}
JG2016-1411	79.93±2.23^{klmno}	2.77±0.03^{hijkl}	143.63±2.21^{b}	198.59±11.42^{kl}	15.01±0.77^{pqrs}	0.33±0.01^{mno}	14.74±0.91^{no}
JG2016-1614	65.47±2.2^{ab}	2.99±0.02^{m}	172.37±1.01^{d}	146.83±8.71^{ab}	10.37±0.73^{abcd}	0.26±0.02^{abc}	9.18±1.29^{ab}
JG2016-36	64.47±0.4^{a}	2.45±0.02^{ef}	204.82±4.32^{g}	150.8±4.57^{abc}	10.39±0.21^{abcd}	0.25±0.01^{ab}	8.79±0.33^{a}
JG2016-44	80.91±1.08^{mno}	2.83±0.01^{l}	190.06±1.53^{f}	220.06±11.78^{m}	16.99±1.2^{t}	0.37±0.02^{p}	15.96±1.37^{o}
JG2016-45	69.15±1.37^{cde}	2.79±0.03^{jkl}	190.32±3.19^{f}	145.32±18.84^{a}	10.26±0.38^{ab}	0.26±0.01^{abc}	9.89±2.25^{abcd}
JG2016-634958	76.12±5.11^{ghij}	2.52±0.02^{f}	194.04±1.34^{f}	188.8±13.39^{ijkl}	14.3±1.68^{nopqr}	0.32±0.03^{lmno}	12.38±1.16^{fghijkl}
JG2016-74315	75.42±2.28^{gh}	2.73±0.01^{ghijk}	228.12±3.31^{kl}	169.63±14.07^{defgh}	12.44±0.86^{fghijk}	0.29±0.02^{ghij}	12.27±1.26^{efghijkl}
JG2016-921814	74.31±2.24^{fg}	2.66±0.01^{g}	231.24±3.47^{lm}	181.3±7.34^{fghij}	13.01±0.57^{ijklmn}	0.3±0.01^{hijk}	12.75±1.1^{ghijklm}
JG2016-9605	70±0.89^{de}	2.2±0.02^{cd}	223.31±3^{k}	161.12±3.68^{abcde}	11.59±0.25^{cdefg}	0.28±0.01^{cdefg}	12.01±0.69^{efghijk}
JG2016-9651	70.18±0.23^{e}	2.97±0.03^{m}	195.2±2.36^{f}	161.51±14.47^{bcde}	11.87±0.1^{efghi}	0.28±0.01^{defgh}	11.07±1.96^{cdefg}
JG2017-48	69.49±1^{cde}	3.21±0.01^{n}	214.83±1.42^{ij}	154.38±3.69^{abcd}	10.89±0.34^{bcde}	0.26±0.01^{bcd}	11.19±0.36^{defg}
JG2018-51	73.75±1.61^{fg}	3.2±0.02^{n}	216.6±2.35^{j}	165.26±2.08^{cdef}	12.43±0.58^{fghijk}	0.29±0.01^{fghij}	10.95±0.3^{bcdefg}
JG2021-1424	71.52±2.07^{ef}	2.49±0.26^{ef}	232.14±3.96^{lmn}	180.23±2.08^{fghij}	13.32±0.37^{jklmno}	0.31±0.04^{ijkl}	13.67±0.53^{jklmn}
JG2021-1617	66.49±2.03^{abcd}	2.81±0.03^{kl}	212.43±2.44^{hij}	154.5±9.65^{abcd}	10.89±0.58^{bcde}	0.27±0.01^{bcde}	9.31±1.1^{abc}
JG2021-6301	68.4±3.21^{bcde}	2.83±0.01^{l}	163.02±4.46^{c}	160.98±8.74^{abcde}	11.67±0.86^{defgh}	0.28±0.02^{cdefg}	10.4±0.96^{abcde}
JG2022-74	66.48±0.6^{abcd}	2.41±0.11^{e}	182.29±2.43^{e}	156.07±3.99^{abcd}	10.33±0.28^{abc}	0.25±0.01^{ab}	8.66±0.28^{a}
JG2022-75	66.03±0.15^{abc}	2.64±0.02^{g}	245.88±3.45^{p}	145.92±1.05^{ab}	9.56±0.22^{a}	0.24±0.01^{a}	8.88±0.89^{a}
JG226	76.71±2.91^{ghijkl}	2.08±0.02^{ab}	171.93±1.56^{d}	182.75±8.04^{ghijkl}	14.11±0.9^{lmnopq}	0.32±0.02^{klmn}	12.16±1.05^{efghijk}
JG24	81.88±1.43^{o}	2.8±0.02^{kl}	236±3.7^{mno}	197.9±5.71^{kl}	15.1±0.63^{qrs}	0.34±0.01^{no}	13.46±0.83^{ijklmn}
JG28	79.06±0.96^{hijklmno}	2.82±0.04^{l}	240±3.85^{o}	186.78±11^{ijkl}	14.33±0.34^{nopqrs}	0.32±0.01^{lmno}	13.8±1.17^{klmn}
JG32	76.48±1.06^{ghijk}	2.7±0.02^{ghij}	235.96±1.69^{mno}	169.84±6.44^{defgh}	12.83±0.31^{ghijkl}	0.3±0.01^{ghijk}	11.7±0.47^{defghi}
JG33	70.85±2.02^{ef}	2.24±0.02^{d}	250.14±2.7^{qr}	155.85±15.14^{abcd}	11.54±0.61^{bcdefg}	0.28±0.01^{cdefg}	10.48±1.01^{abcdef}

JG36	86.71±3.93^{p}	2.82±0.02^{l}	143.28±1.65^{b}	218.21±5.42^{m}	17.3±1.23^{t}	0.37±0.02^{p}	16.13±0.68^{o}
JG42	81.11±1.03^{no}	2.69±0.04^{ghi}	173.27±2.89^{d}	188.16±6.5^{ijkl}	14.11±0.37^{lmnopq}	0.32±0.01^{klmn}	14.16±0.67^{lmn}
JG6	77.11±1.77^{ghijklm}	2.06±0.01^{ab}	145.17±2.48^{b}	170.05±3.62^{defgh}	12.97±0.43^{hijklm}	0.3±0.01^{ghijk}	11.2±0.68^{defg}
JG63	81.05±2.13^{no}	3.58±0.03^{q}	111.19±1.75^{a}	192.13±5.08^{jkl}	14.72±1.09^{pqrs}	0.33±0.02^{lmno}	13.42±0.54^{ijklmn}
JG74	77.41±2.13^{ghijklmn}	2.02±0.01^{a}	205.44±4.24^{g}	182.54±2.17^{ghijk}	14.52±0.76^{opqrs}	0.33±0.02^{lmno}	13.29±0.29^{hijklmn}
PG205	79.29±2.16^{ijklmno}	2.82±0.01^{l}	239.79±3.44^{o}	199.03±6.02^{l}	15.59±0.34^{rs}	0.34±0.01^{o}	13.82±1.33^{klmn}
**Mean**	**75.2±5.81**	**2.79±0.44**	**203.92±34.76**	**176.42±20.49**	**13.23±1.99**	**0.3±0.03**	**12.26±2.04**

Data pooled for two successive seasons and presented as mean of triplicate ± standard deviation. Means with the same letter are not significantly different at 5% level of significance

Supplementary Table S3 Pooled physiological responses of various chickpea genotypes under drought stress at vegetative stage

Genotypes	RWC	CTD	SLA	Ci	Photo	Conduct	Trmmol
ICC4958	71.24±2.85^{pqr}	0.54±0.01^{k}	140.83±1.46^{gh}	137.02±7.11^{hijklm}	8.77±0.92^{jklmn}	0.22±0.02^{ijklm}	10±0.76^{jklmno}
ICCV15102	61.5±1.28^{hijk}	0.28±0.02^{gh}	181.69±2.35^{o}	120.87±1.39^{defg}	7.13±0.43^{defgh}	0.19±0.01^{cdefg}	8.17±0.28^{efg}
ICCV15115	70.37±0.11^{opq}	0.23±0.05^{fg}	163.82±1.05^{lm}	145.04±4.1^{klmno}	9.88±0.74^{nop}	0.24±0.01^{mno}	10.67±0.69^{nopq}
ICCV15118	67.59±2.26^{mnop}	0.26±0.03^{fgh}	181.09±2.04^{o}	134.01±5.79^{ghijkl}	9.72±0.72^{nop}	0.23±0.02^{lmno}	10.31±0.63^{lmnop}
ICCV181664	70.16±1.9^{op}	0.12±0.02^{e}	181.67±2.41^{o}	154.6±7.76^{no}	10.53±0.79^{pq}	0.25±0.02^{op}	11.21±0.77^{opqr}
ICCV19616	57.49±2.36^{defg}	0.51±0.05^{jk}	142.44±1.64^{gh}	110.71±4.79^{abcd}	6.47±0.62^{bcdef}	0.18±0.03^{bcde}	7.88±0.22^{cdef}
JAKI9218	74.44±0.78^{r}	0.56±0.02^{k}	158.47±2.07^{j}	153.16±0.72^{no}	10.23±0.59^{opq}	0.24±0.01^{nop}	11.04±0.4^{nopq}
JG11	74.72±1.01^{r}	0.29±0.03^{h}	159.11±1.25^{jk}	150.22±1.86^{mno}	10.25±0.71^{opq}	0.24±0.02^{nop}	11.3±0.68^{pqr}
JG14	58.59±1.76^{fgh}	-0.35±0.02^{b}	186.96±3.43^{pq}	125.61±5.97^{efghi}	8.39±0.48^{ijklm}	0.21±0.01^{hijkl}	8.97±0.44^{fghijk}
JG16	71.54±1.56^{pqr}	0.37±0.03^{i}	119.29±0.35^{d}	140.54±3.71^{jklmn}	9.95±0.36^{nop}	0.24±0.01^{mno}	10.62±0.32^{nopq}
JG17	59.31±1.36^{fgh}	0.21±0.01^{f}	170.68±4.56^{n}	119.74±3.09^{cdefg}	7.01±0.24^{cdefgh}	0.19±0.01^{bcdef}	8.31±0.28^{efgh}
JG2003-14-16	68.71±1.58^{nop}	0.13±0.04^{e}	199.03±4.42^{s}	141.23±14.09^{klmn}	9.76±0.91^{nop}	0.23±0.02^{lmno}	10.42±0.84^{mnop}
JG2016-1411	66.7±1.98^{lmno}	0.06±0.03^{d}	105.76±0.65^{b}	153.14±10.02^{no}	9.48±0.71^{mnop}	0.23±0.01^{lmno}	10.34±0.79^{lmnop}
JG2016-1614	53.24±2.86^{abc}	0.36±0.01^{i}	144.55±2.49^{h}	105.05±8.14^{ab}	5.96±0.71^{abcde}	0.17±0.04^{abc}	6.81±0.76^{abc}
JG2016-36	50.47±2.03^{a}	-0.11±0.01^{c}	182.49±4.55^{op}	109.32±3.34^{abcd}	5.91±0.29^{abcd}	0.17±0.01^{abcd}	6.62±0.29^{ab}
JG2016-44	70.99±1.43^{pqr}	0.05±0^{d}	138.46±1.27^{fg}	168.97±15.53^{p}	11.82±1.02^{r}	0.27±0.02^{q}	12.26±0.98^{r}
JG2016-45	56.83±1.67^{cdef}	0.23±0.02^{fg}	160.68±2.28^{jkl}	105.56±6.84^{abc}	5.72±0.42^{ab}	0.17±0.01^{ab}	6.58±0.28^{ab}
JG2016-634958	64.56±5.45^{jklm}	0.12±0.02^{e}	163.12±2.36^{klm}	150.18±18.84^{mno}	9.56±1.6^{mnop}	0.23±0.03^{lmno}	10.13±1.51^{klmnop}
JG2016-74315	61.88±2.92^{hijk}	0.41±0.01^{i}	180.11±2.7^{o}	126.91±7.17^{efghij}	6.86±0.74^{bcdefg}	0.19±0.02^{bcdef}	8.05±0.64^{defg}
JG2016-921814	61.09±2.2^{ghij}	0.04±0.03^{d}	180.8±3.96^{o}	126.43±2.92^{efghij}	8.06±0.46^{ghijk}	0.21±0.01^{ghijk}	8.81±0.41^{fghi}
JG2016-9605	51.1±0.71^{ab}	-0.33±0.01^{b}	189.65±2.72^{q}	112.41±6.97^{abcde}	5.84±0.23^{abc}	0.17±0.01^{abc}	6.6±0.22^{ab}
JG2016-9651	56.97±0.7^{cdef}	0.24±0.01^{fg}	172.59±2.54^{n}	122.56±9.86^{defgh}	7.13±0.01^{defgh}	0.19±0.01^{cdefg}	8.02±0.18^{defg}
JG2017-48	58.39±1.61^{efgh}	0.6±0.02^{l}	166.26±2.55^{m}	104.97±9.11^{ab}	5.98±0.46^{abcde}	0.17±0.03^{abc}	6.95±0.58^{abcd}
JG2018-51	65.05±1.99^{jklmn}	0.64±0.01^{l}	186.98±0.75^{pq}	126.1±6.95^{efghij}	7.87±0.64^{ghij}	0.2±0.01^{efghi}	9.42±0.89^{hijklm}
JG2021-1424	56.27±1.99^{cdef}	0.29±0.02^{h}	196.04±2.01^{rs}	123.43±2.99^{defghi}	7.65±0.26^{fghij}	0.2±0.001^{efghi}	8.4±0.29^{efgh}
JG2021-1617	53.48±2.64^{abcd}	0.14±0.01^{e}	180.12±1.59^{o}	118.75±8.18^{bcdef}	6.32±0.67^{bcde}	0.18±0.01^{abcd}	7.45±0.58^{bcde}
JG2021-6301	55.86±2.83^{cdef}	0.22±0.01^{f}	140.27±3.47^{fgh}	121.84±12.91^{defg}	7.22±0.77^{efghi}	0.19±0.02^{defgh}	8.08±0.8^{defg}
JG2022-74	54.46±0.43^{bcde}	-0.11±0.02^{c}	131.82±2.37^{e}	112.31±6.72^{abcde}	5.97±0.27^{abcde}	0.17±0.01^{abcd}	6.71±0.28^{ab}
JG2022-75	53.48±0.94^{abcd}	-0.32±0.01^{b}	182.73±3.17^{op}	104.01±0.96^{a}	5.06±0.26^{a}	0.16±0.01^{a}	5.89±0.18^{a}
JG226	64.14±2.97^{jklm}	-0.35±0.01^{ab}	131.96±1.14^{e}	147.23±6.2^{lmno}	9.53±0.83^{mnop}	0.23±0.02^{lmno}	10.08±0.74^{klmno}
JG24	71.45±0.65^{pqr}	0.21±0.05^{f}	194.05±4.35^{r}	154.89±3.23^{no}	10.33±0.61^{opq}	0.25±0.01^{opq}	11.14±0.61^{opqr}
JG28	65.39±1.43^{klmn}	0.27±0.02^{gh}	188.64±3.58^{q}	137.76±10.16^{ijklm}	9.15±0.41^{klmno}	0.22±0.01^{jklmn}	9.85±0.57^{ijklmn}
JG32	64.35±1.52^{jklm}	0.5±0.02^{j}	181.34±2.34^{o}	130.87±3.93^{fghijk}	8.24±0.35^{hijkl}	0.21±0.02^{ghijk}	9.17±0.39^{ghijkl}
JG33	59.41±3.64^{fghi}	0.06±0.06^{d}	198.08±2.34^{rs}	117.59±9.3^{abcdef}	7.05±0.72^{cdefgh}	0.19±0.01^{cdefg}	8.03±0.8^{defg}

JG36	64.36±1.4^{jklm}	0.36±0.01^{i}	110.86±1.56^{c}	148.58±5.6^{mno}	9.61±0.27^{mnop}	0.23±0.01^{lmno}	10.48±0.4^{mnop}
JG42	63.91±0.96^{jklm}	0.15±0.05^{e}	136.25±2.63^{f}	131.49±2.09^{fghijk}	8.04±0.39^{ghijk}	0.21±0.04^{fghij}	8.9±0.34^{fghij}
JG6	63.32±1.79^{ijkl}	-0.32±0.01^{b}	110.04±2.32^{c}	125.91±2.64^{efghi}	8.2±0.44^{hijkl}	0.21±0.01^{fghij}	8.76±0.42^{fghi}
JG63	74.14±2.97^{qr}	0.55±0.05^{k}	90.93±1.8^{a}	145.24±3.19^{klmno}	10.26±1.18^{opq}	0.24±0.02^{nop}	10.97±0.91^{nopq}
JG74	61.6±3.1^{hijk}	-0.39±0.02^{a}	150.59±2.15^{i}	142.52±7.88^{klmno}	9.37±0.75^{lmnop}	0.23±0.01^{klmno}	9.91±0.69^{ijklmn}
PG205	69.48±1.79^{op}	0.13±0.05^{e}	166.17±1.86^{m}	156.32±6.68^{o}	11.29±0.32^{qr}	0.26±0.01^{pq}	11.75±0.38^{qr}
**Mean**	**62.95±7.01**	**0.17±0.28**	**161.16±28.17**	**131.58±18.13**	**8.29±1.83**	**0.21±0.03**	**9.13±1.73**

Data pooled for two successive seasons and presented as mean of triplicate ± standard deviation. Means with the same letter are not significantly different at 5% level of significance

Supplementary Table S4 Pooled biochemical responses of various chickpea genotypes under normal irrigated condition

**Genotypes**	**Chla**	**Chlb**	**Protein**	**H_2O_2**	**EL (%)**	**MDA**	**Sugar**	**Proline**
ICC4958	0.45±0.02^{def}	0.38±0.03^{mno}	0.42±0.03^{b}	1.68±0.02^{cd}	33.82±0.52^{abc}	5.64±0.08^{p}	1.43±0.05st	30.78±0.19^{n}
ICCV15102	0.45±0.01^{def}	0.34±0.06^{h}	0.45±0.02^{ef}	1.76±0.04^{efghi}	40.26±1.47^{nopq}	7.6±0.03^{yz}	1.27±0.02^{mn}	34.69±0.11^{s}
ICCV15115	0.45±0.02^{def}	0.37±0.02^{lmn}	0.45±0.03^{fg}	1.95±0.04^{opqrs}	37.49±0.37^{hijklm}	2.11±0.04^{c}	1.23±0.04^{ijk}	23.06±0.28^{g}
ICCV15118	0.45±0.03^{def}	0.33±0.03^{h}	0.45±0.01^{ef}	1.83±0.04^{hijklm}	38.59±0.51^{klmn}	4.95±0.05^{m}	1.27±0.04^{no}	22.86±0.11^{fg}
ICCV181664	0.44±0.02^{cd}	0.33±0.02^{h}	0.47±0.02^{jk}	1.97±0.04^{pqrs}	40.46±0.72^{nopq}	7.43±0.06^{x}	1.24±0.04^{jkl}	30.8±0.22^{n}
ICCV19616	0.46±0.04^{fgh}	0.3±0.01^{ef}	0.45±0.03^{fg}	1.88±0.01^{jklmno}	39.12±1.07^{mnop}	7.15±0.02^{w}	1.15±0.03^{e}	35.7±0^{t}
JAKI9218	0.46±0.03^{gh}	0.37±0.03^{lm}	0.41±0.03^{a}	1.56±0.02^{a}	35.18±0.49^{abcdef}	7.07±0.04^{w}	1.40±0.04^{r}	32.12±0^{p}
JG11	0.46±0.03^{fgh}	0.36±0.03^{kl}	0.44±0.06^{d}	1.6±0.05^{ab}	34.53±0.31^{abcde}	6.9±0.26^{v}	1.41±0.08^{rs}	30.15±0.11^{m}
JG14	0.45±0.02^{def}	0.23±0.04^{c}	0.47±0.02^{jk}	1.92±0.03^{nopq}	37.18±1.24^{ghijkl}	4.34±0.11^{k}	1.22±0.04^{i}	22.72±0.57^{fg}
JG16	0.48±0.03^{ij}	0.32±0.03^{g}	0.42±0.02^{b}	1.64±0.03^{bc}	35.25±0.75^{abcdefg}	8.04±0.3^{z1}	1.19±0.03^{gh}	34.41±0.33^{s}
JG17	0.47±0.05^{ij}	0.19±0.02^{b}	0.44±0.03^{de}	1.84±0.03^{ijklmn}	36.91±0.4^{fghijk}	4.59±0.02^{l}	1.29±0.06^{o}	21.33±0.22^{cd}
JG2003-14-16	0.42±0.02^{b}	0.3±0.01^{ef}	0.46±0.05^{fgh}	1.94±0.05^{opqr}	37.5±1.47^{hijklm}	3.81±0.04^{i}	1.18±0.03^{gh}	30.68±0.29^{n}
JG2016-1411	0.45±0.03^{efg}	0.31±0.05^{g}	0.45±0.03^{fg}	1.94±0.05^{opqr}	37.58±0.64^{ijklm}	1.37±0.08^{a}	1.29±0.05^{o}	19.04±0.11^{a}
JG2016-1614	0.44±0.06^{de}	0.38±0.02^{no}	0.46±0.02^{ghi}	2.02±0.04^{rs}	35.97±0.33^{efghij}	3.66±0.05^{h}	1.22±0.04^{i}	21.27±0.19^{cd}
JG2016-36	0.45±0.03^{de}	0.37±0.03^{lm}	0.50±0.04^{n}	2.03±0.01^{s}	41.55±1.92^{q}	4.35±0.01^{k}	1.26±0.03^{lmn}	19.91±0^{b}
JG2016-44	0.45±0.03^{de}	0.34±0.02^{hi}	0.47±0.02^{jk}	2±0.04^{qrs}	38.89±0.96^{lmno}	7.74±0.02^{z}	1.22±0.04^{i}	36.08±0^{t}
JG2016-45	0.46±0.01^{gh}	0.34±0.04^{h}	0.45±0.02^{ef}	1.8±0.03^{fghij}	37.63±1.81^{jklm}	5.05±0.11^{n}	1.24±0.04^{ijk}	29.28±0.23^{l}
JG2016-634958	0.46±0.03^{gh}	0.29±0.04^{d}	0.47±0.03^{k}	1.96±0.03^{pqrs}	33.93±0.96^{abcd}	6.14±0.01^{s}	1.47±0.05^{u}	24.49±0^{i}
JG2016-74315	0.46±0.02^{fgh}	0.37±0.03^{lm}	0.46±0.02^{ghi}	1.81±0.05^{fghij}	35.67±1.49^{cdefghi}	3.46±0.05^{g}	1.17±0.04^{fg}	26.2±0^{j}
JG2016-921814	0.44±0.05^{cd}	0.38±0.03^{o}	0.45±0.04^{ef}	1.84±0.05^{ijklmn}	33.67±0.71^{ab}	5.02±0.02^{m}	1.43±0.04st	29.77±0.11^{m}
JG2016-9605	0.47±0.02^{hi}	0.35±0.04^{jk}	0.44±0.02^{d}	1.98±0.06^{pqrs}	35.4±1.28^{bcdefg}	4.62±0.22^{lm}	1.04±0.03^{a}	42.75±0.95^{u}
JG2016-9651	0.45±0.02^{def}	0.31±0.02^{fg}	0.52±0.03^{p}	1.95±0.05^{opqrs}	35.55±0.92^{bcdefgh}	4.13±0.05^{j}	1.33±0.04^{p}	21.58±0^{d}
JG2017-48	0.45±0.03^{efg}	0.33±0.02^{h}	0.49±0.02^{m}	1.9±0.02^{lmnop}	38.93±0.2^{lmnop}	5.97±0.02^{qr}	1.46±0.05^{u}	31.1±0.11^{n}
JG2018-51	0.45±0.01^{efg}	0.31±0.03^{fg}	0.48±0.02^{l}	2.21±0.03^{u}	39.2±0.58^{mnop}	5.36±0.01^{o}	1.36±0.04^{q}	24.49±0^{i}
JG2021-1424	0.44±0.03^{de}	0.38±0.04^{mno}	0.53±0.03^{q}	2.02±0.06^{rs}	35.05±0.3^{abcdef}	3.07±0.07^{f}	1.19±0.04^{h}	19.84±0.3^{b}
JG2021-1617	0.44±0.02^{cd}	0.33±0.02^{h}	0.51±0.02^{o}	2.12±0.1^{t}	33.42±0.65^{a}	7.55±0.01^{xy}	1.23±0.03^{ij}	33.45±0^{r}
JG2021-6301	0.45±0.02^{def}	0.36±0.03^{kl}	0.58±0.03^{r}	2.13±0.06^{t}	34.15±0.27^{abcde}	2.69±0.08^{e}	1.48±0.06^{u}	22.52±0.28^{ef}
JG2022-74	0.44±0.03^{de}	0.37±0.05^{lm}	0.44±0.02^{d}	1.76±0.05^{defgh}	35.46±1.86^{bcdefg}	2.98±0.06^{f}	1.15±0.04^{de}	21.11±0.28^{cd}
JG2022-75	0.45±0.03^{efg}	0.31±0.03^{fg}	0.52±0.05^{p}	1.97±0.03^{pqrs}	39.9±2.45^{nopq}	6.33±0.06^{t}	1.09±0.04^{b}	27.6±0.29^{k}
JG226	0.38±0.06^{a}	0.37±0.03^{lmno}	0.47±0.02^{jk}	1.82±0.06^{ghijk}	40.76±0.43^{opq}	2.32±0.02^{d}	1.25±0.03^{klm}	22.15±0^{e}
JG24	0.46±0.03^{gh}	0.24±0.03^{c}	0.44±0.02^{d}	1.74±0.03^{defg}	36.52±0.62^{fghij}	4.64±0.15^{lm}	1.16±0.03^{ef}	21.07±0.67^{c}
JG28	0.48±0.02^{j}	0.17±0.01^{a}	0.45±0.02^{ef}	1.72±0.07^{cde}	36.68±0.64^{fghij}	4.34±0.03^{k}	1.13±0.04^{cd}	23.81±0.22^{h}
JG32	0.46±0.03^{gh}	0.29±0.03^{de}	0.45±0.03^{fg}	1.73±0.04^{def}	36.58±0.79^{fghij}	5.33±0.08^{o}	1.32±0.05^{p}	26.6±0.22^{j}
JG33	0.45±0.03^{efg}	0.3±0.02^{ef}	0.46±0.02^{ghi}	1.9±0.05^{klmnop}	36.66±0.27^{fghij}	1.79±0.03^{b}	1.37±0.04^{q}	22.48±0.11^{ef}

JG36	0.43±0.03^{c}	0.35±0.04^{ij}	0.46±0.03^{hij}	1.9±0.04^{klmnop}	39.99±0.6^{nopq}	6.16±0.1^{s}	1.12±0.04^{c}	32.93±0.48^{q}
JG42	0.46±0.02^{fgh}	0.38±0.03^{mno}	0.47±0.03^{ijk}	1.91±0.01^{mnop}	40.81±0.93^{pq}	6.6±0.04^{u}	1.15±0.03^{e}	34.62±0.22^{s}
JG6	0.45±0.03^{efg}	0.31±0.02^{fg}	0.42±0.02^{b}	1.56±0.03^{a}	36.92±1.17^{fghijk}	5.88±0.03^{q}	1.29±0.06^{o}	31.55±0^{o}
JG63	0.45±0.05^{def}	0.33±0.02^{h}	0.42±0.06^{b}	1.7±0.02^{cde}	35.62±0.38^{cdefgh}	4.52±0.06^{l}	1.44±0.04^{t}	29.09±0.3^{l}
JG74	0.44±0.03^{de}	0.35±0.06^{ij}	0.43±0.02^{c}	1.8±0.05^{fghij}	35.79±0.67^{defghij}	6.1±0.05^{rs}	1.34±0.04^{p}	32.1±0.19^{p}
PG205	0.44±0.02^{cd}	0.32±0.03^{g}	0.46±0.03^{fgh}	1.82±0.05^{hijkl}	40.54±0.64^{opq}	4.77±0.02^{m}	1.1±0.04^{b}	19.86±0^{b}
**Mean**	**0.45±0.04**	**0.33±0.06**	**0.46±0.05**	**1.86±0.15**	**37.13±2.41**	**5.04±1.74**	**1.26±0.12**	**27.4±5.79**

Data pooled for two successive seasons and presented as mean of triplicate ± standard deviation. Means with the same letter are not significantly different at 5% probability level

Supplementary Table S5 Pooled biochemical responses of various chickpea genotypes under drought stressat vegetative stage

**Genotypes**	**Chla**	**Chlb**	**Protein**	**H_2O_2**	**EL (%)**	**MDA**	**Sugar**	**Proline**
ICC4958	0.34±0.03^{mn}	0.21±0.04^{r}	0.20±0.04^{fgh}	2.57±0.03^{hij}	43.76±0.52^{a}	6.99±0.09^{j}	1.66±0.04^{rs}	66.83±0.67^{s}
ICCV15102	0.32±0.04^{ij}	0.14±0.02^{lm}	0.19±0.02^{d}	2.44±0.05^{cde}	50.63±4.87^{bc}	10.52±0.07^{q}	1.43±0.04^{kl}	49.64±0.23^{p}
ICCV15115	0.31±0.02^{ef}	0.16±0.01^{op}	0.21±0.03^{ghi}	2.46±0.03^{cdef}	53.97±2.83^{fghijkl}	2.97±0.04^{b}	1.42±0.08^{ijkl}	41.33±0.88^{ijklmn}
ICCV15118	0.32±0.06^{hi}	0.15±0.04^{nop}	0.22±0.02^{kl}	2.73±0.04^{lmnop}	52.61±1.68^{cdefghi}	7.13±0.03^{jk}	1.4±0.04^{hij}	40.3±0.1^{ghi}
ICCV181664	0.32±0.03^{hi}	0.14±0.02^{lm}	0.22±0.03^{l}	2.53±0.03^{ghij}	54.72±1.2^{hijklmn}	10.19±0.02^{p}	1.43±0.04^{jkl}	41.37±0.3^{ijklmn}
ICCV19616	0.31±0.02^{ef}	0.12±0.03^{ij}	0.22±0.02^{kl}	2.47±0.05^{defg}	54.48±0.51^{hijklm}	10.89±0.1^{r}	1.33±0.04^{def}	49.2±0.43^{op}
JAKI9218	0.33±0.01^{lm}	0.21±0.02^{r}	0.18±0.04^{c}	2.39±0.02^{abc}	45.31±0.27^{a}	8.72±0.18^{m}	1.64±0.04^{qr}	67.53±1.17^{s}
JG11	0.34±0.03^{mn}	0.21±0.05^{r}	0.21±0.02^{hij}	2.46±0.04^{cdef}	44.69±0.38^{a}	9.78±0.15^{o}	1.66±0.04^{rs}	69.91±0.6^{t}
JG14	0.31±0.02^{ef}	0.06±0.02^{d}	0.20±0.01^{efg}	2.68±0.04^{lm}	57.03±0.31^{mnop}	6.5±0.07^{i}	1.39±0.05^{h}	38.52±0.4^{def}
JG16	0.35±0.04^{o}	0.14±0.01^{kl}	0.20±0.02^{efg}	2.56±0.04^{hij}	51.72±0.23^{bcdefg}	10.33±0.13^{pq}	1.40±0.05^{hij}	69.83±0.61^{t}
JG17	0.33±0.03^{lm}	0.02±0.03^{b}	0.20±0.04^{efg}	2.7±0.01^{lmno}	53.95±0.12^{fghijkl}	7.12±0.04^{j}	1.45±0.06^{l}	36.53±0.15^{bc}
JG2003-14-16	0.27±0.03^{b}	0.12±0.02^{i}	0.19±0.02^{d}	2.35±0.02^{ab}	53.1±1.06^{cdefghij}	5.49±0.14^{g}	1.34±0.05^{def}	42.02±0.68^{klmn}
JG2016-1411	0.32±0.02^{hi}	0.13±0.03^{kl}	0.20±0.03^{def}	2.46±0.01^{defg}	55.83±0.73^{klmno}	2.32±0.09^{a}	1.42±0.06^{ijkl}	36.23±0.31^{abc}
JG2016-1614	0.32±0.02^{hi}	0.15±0.02^{no}	0.21±0.03^{ijk}	2.41±0.03^{bcd}	51.21±1.17^{bcde}	6.42±0.09^{i}	1.38±0.03^{gh}	38.17±0.62^{de}
JG2016-36	0.33±0.03^{jk}	0.15±0.03^{mn}	0.24±0.06^{n}	2.72±0.06^{lmnop}	53.35±1.1^{defghijk}	7.32±6.12^{k}	1.43±0.05^{kl}	36.35±0.3^{abc}
JG2016-44	0.32±0.01^{hi}	0.16±0.06^{p}	0.24±0.02^{n}	2.77±0.06^{op}	54.86±0.92^{hijklmn}	10.77±0.09^{r}	1.39±0.04^{hi}	48.09±0.36^{o}
JG2016-45	0.33±0.02^{kl}	0.12±0.03^{hi}	0.19±0.02^{d}	2.59±0.04^{jk}	55.14±1.22^{ijklmn}	7.82±0.09^{l}	1.43±0.04^{jkl}	41.65±0.35^{jklmn}
JG2016-634958	0.31±0.05^{ef}	0.11±0.02^{h}	0.22±0.02^{kl}	2.51±0.04^{efgh}	61.02±0.92^{q}	9.74±0.09^{o}	1.68±0.06^{s}	40.89±0.59^{hijklm}
JG2016-74315	0.31±0.02^{ef}	0.13±0.06^{jk}	0.22±0.03^{jkl}	2.6±0.04^{jk}	53.33±0.83^{defghijk}	5.23±0.03^{f}	1.35±0.03^{f}	41.85±0.2^{klmn}
JG2016-921814	0.31±0.03^{e}	0.16±0.02^{p}	0.19±0.03^{de}	2.51±0.05^{fghi}	58.56±0.87^{p}	7.95±0.14^{l}	1.60±0.05^{p}	41.85±0.77^{klmn}
JG2016-9605	0.32±0.02^{hi}	0.13±0.04^{jk}	0.16±0.03^{b}	2.36±0.03^{ab}	53±1.59^{cdefghij}	9.07±0.27^{n}	1.17±0.04^{a}	52.17±0.77^{q}
JG2016-9651	0.31±0.02^{ef}	0.13±0.02^{jk}	0.25±0.02^{n}	2.76±0.06^{nop}	57.17±1.51^{nop}	7.02±0.17^{j}	1.49±0.03^{mn}	35.96±0.13^{ab}
JG2017-48	0.31±0.03^{fg}	0.15±0.02^{no}	0.24±0.05^{n}	2.68±0.03^{lm}	53.91±1.33^{fghijkl}	8±0.08^{l}	1.63±0.09^{pq}	38.6±0.4^{ef}
JG2018-51	0.32±0.02^{hi}	0.14±0.01^{lm}	0.25±0.02^{n}	2.69±0.04^{lmn}	53.67±1.06^{efghijkl}	7.96±0.08^{l}	1.57±0.04^{o}	41.27±0.39^{ijklmn}
JG2021-1424	0.31±0.02^{ef}	0.16±0.02^{p}	0.27±0.02^{o}	2.74±0.03^{lmnop}	52.78±1.06^{cdefghi}	5.29±0.11^{fg}	1.36±0.04^{fg}	37.96±0.3^{de}
JG2021-1617	0.32±0.03^{hi}	0.14±0.04^{lm}	0.27±0.02^{o}	3.05±0.05^{s}	52.24±0.57^{cdefgh}	11.88±0.13^{t}	1.39±0.06^{gh}	42.16±1.1^{lmn}
JG2021-6301	0.31±0.02^{ef}	0.16±0.02^{p}	0.30±0.03^{p}	2.85±0.04^{q}	54.39±0.7^{hijklm}	4.83±0.14^{e}	1.65±0.04^{qr}	42.27±0.41^{n}
JG2022-74	0.31±0.04^{ef}	0.15±0.02^{no}	0.22±0.03^{l}	2.64±0.04^{kl}	51.47±1.45^{bcdef}	4.55±0.13^{d}	1.3±0.03^{c}	40.88±0.75^{hijkl}
JG2022-75	0.32±0.02^{hi}	0.13±0.01^{jk}	0.27±0.02^{o}	2.78±0.05^{p}	50.8±0.6^{bcd}	8.68±0.07^{m}	1.22±0.04^{b}	35.24±0.23^{a}
JG226	0.24±0.02^{a}	0.14±0.02^{lm}	0.19±0.03^{de}	2.48±0.03^{defg}	55.31±0.51^{jklmn}	4.09±0.12^{c}	1.41±0.04^{hijk}	41.36±0.99^{ijklmn}
JG24	0.33±0.03^{jk}	0.05±0.03^{c}	0.19±0.02^{d}	2.42±0.03^{bcd}	56.03±0.64^{lmno}	7.02±0.1^{j}	1.31±0.04^{cde}	41.78±0.58^{klmn}
JG28	0.34±0.02^{n}	0.01±0.02^{a}	0.19±0.05^{d}	2.49±0.06^{efg}	49.5±0.02^{b}	6.94±0.18^{j}	1.30±0.03^{c}	42.25±1.03^{mn}
JG32	0.32±0.01^{hi}	0.08±0.04^{e}	0.19±0.02^{d}	2.35±0.06^{ab}	52.56±0.32^{cdefghi}	7.79±0.11^{l}	1.5±0.05^{mn}	41.61±0.5^{ijklmn}
JG33	0.32±0.03^{ij}	0.09±0.02^{f}	0.20±0.02^{efg}	2.72±0.03^{lmnop}	55.19±0.23^{ijklmn}	2.87±0.02^{b}	1.55±0.05^{o}	40.36±1.66^{ghij}

JG36	0.32±0.02^{hi}	0.12±0.02^{i}	0.21±0.02^{hij}	2.76±0^{nop}	55.3±2.1^{jklmn}	9.04±0.16^{n}	1.31±0.04^{cd}	39.83±0.83^{gh}
JG42	0.32±0.03^{gh}	0.14±0.01^{lm}	0.22±0.04^{kl}	2.58±0.02^{ijk}	54.11±1.18^{ghijklm}	9.16±0.14^{n}	1.34±0.04^{def}	41.78±0.97^{klmn}
JG6	0.30±0.02^{d}	0.10±0.05^{g}	0.12±0.02^{a}	2.34±0.03^{a}	58.11±0.57^{op}	11.66±0.08^{s}	1.48±0.06^{m}	39.52±0.29^{fg}
JG63	0.33±0.02^{kl}	0.18±0.02^{q}	0.21±0.02^{hij}	2.93±0.04^{r}	45.68±0.55^{a}	5.69±0.15^{h}	1.66±0.05^{rs}	60.73±0.84^{r}
JG74	0.29±0.04^{c}	0.10±0.02^{g}	0.16±0.03^{b}	2.45±0.03^{cdef}	58.68±0.52^{p}	13.14±0.04^{u}	1.51±0.05^{n}	37.32±0.46^{cd}
PG205	0.32±0.02^{hi}	0.14±0.02^{lm}	0.22±0.03^{jkl}	2.7±0.04^{lmno}	52.89±2.34^{cdefghij}	6.45±0.22^{i}	1.25±0.05^{b}	40.69±1.54^{ghijk}
**Mean**	**0.32±0.04**	**0.13±0.05**	**0.21±0.05**	**2.59±0.17**	**53.3±3.83**	**7.63±2.72**	**1.44±0.13**	**44.3±9.4**

Data pooled for two successive seasons and presented as mean of triplicate ± standard deviation. Means with the same letter are not significantly different at 5% probability level

Supplementary Table S6 Pooled yield and its attributing trait responses of various chickpea genotypes under normal irrigated condition

**Genotypes**	**NOP**	**SY**	**BY**	**HI**	**100-SW**	**DTF**	**DTM**
ICC4958	43.15±1.95^{ghi}	3.53±0.23^{klmn}	26.93±1.11^{ijklmn}	35.32±1.15^{efghij}	29.75±0.24^{vw}	57.08±0.52^{bc}	103.83±1.26^{bcde}
ICCV15102	33.53±1.01^{bc}	2.66±0.11^{abcd}	29.54±0.49^{no}	31.26±0.94^{abcde}	24.86±0.55^{t}	63.91±0.37^{ijklmno}	110.5±1^{klmn}
ICCV15115	30.49±2.26^{ab}	3.38±0.1^{ijkl}	29.58±2.23^{no}	31.35±2.53^{abcdef}	22.92±0.67^{rs}	63.63±0.34^{hijklmn}	105.5±1^{cdefgh}
ICCV15118	27.17±1.88^{a}	3.35±0.09^{hijkl}	29.59±0.18^{no}	30.72±1.24^{abcde}	20.96±1.3^{mnop}	64.83±1.53^{klmnopqr}	104.83±0.29^{cdefg}
ICCV181664	29.94±4.31^{ab}	3.14±0.21^{efghijkl}	21.57±0.24^{ab}	40.84±0.46^{klm}	21.16±0.1^{nop}	66.36±0.99^{pqrs}	107.17±0.76^{efghij}
ICCV19616	36.56±5.58^{cde}	3.88±0.09^{n}	21.06±1.22^{a}	41.23±1.69^{lm}	23.31±1.24^{s}	64.77±1.92^{klmnopq}	107±1.32^{efghij}
JAKI9218	60.22±1.61^{k}	4.70±0.37^{op}	27.98±0.3^{klmno}	40.96±2.66^{klm}	28.71±0.29^{v}	60±0.25^{de}	106±1.73^{defgh}
JG11	52.13±2.41^{j}	4.82±0.35^{p}	26.78±0.86^{hijklmn}	41.43±1.2^{lm}	25.64±0.34^{tu}	55.75±2.17^{ab}	104±0.87^{bcde}
JG14	26.44±0.96^{a}	2.32±0.06^{a}	24.45±1.03^{cdefghi}	34.36±3.9^{defghij}	20.29±0.16^{klmn}	61.5±0.66^{efgh}	104±1.73^{bcde}
JG16	58.5±1.32^{k}	4.34±0.06^{o}	39.60±1.46^{p}	29.19±1.13^{abc}	26.72±0.2^{u}	61.92±1.38^{efghi}	111.5±2.5^{mn}
JG17	29.4±1.46^{ab}	3.11±0.14^{cdefghijk}	24.10±1.32^{bcdefghi}	31.99±5.45^{bcdef}	19.41±0.2^{hijkl}	59.75±0.43^{de}	103.5±4.36^{abcd}
JG2003-14-16	42.38±3.96^{ghi}	3.23±0.14^{hijkl}	27.88±1.1^{klmno}	32.61±1.56^{bcdefg}	23.42±0.82^{s}	59.78±0.71^{de}	100.5±1.8^{a}
JG2016-1411	50.07±2.08^{j}	3.20±0.31^{ghijkl}	23.51±0.49^{abcdef}	38.07±1.24^{hijkl}	17.43±1.27^{cde}	59.02±1.33^{cd}	106.5±2.18^{defghi}
JG2016-1614	28.06±2.19^{a}	3.19±0.16^{ghijkl}	26.50±1.64^{ghijklm}	32.17±1.89^{bcdefg}	15.45±1^{b}	64.99±1.29^{lmnopqr}	101.17±1.26^{ab}
JG2016-36	39.96±3.11^{efg}	3.45±0.23^{jiklm}	24.74±0.83^{cdefghijk}	35.55±1.17^{efgihj}	16.93±0.2^{c}	65.39±1.42^{mnopqrs}	110.83±1.04^{lmn}
JG2016-44	26.1±1.41^{a}	3.06±0.25^{cdefghij}	27.62±1.17^{jklmn}	31.54±1.88^{abcdef}	17.96±0.27^{cdefg}	64.69±0.41^{jklmnop}	105±0.87^{cdefg}
JG2016-45	36.55±3.64^{cde}	3.82±0.36^{lmn}	25.15±1.35^{cdefghijk}	34.91±2.7^{defghij}	17.91±0.47^{cdefg}	62.97±0.46^{ghijkl}	104.83±3.18^{cdefg}
JG2016-634958	27.31±3.32^{a}	3.19±0.35^{fghijkl}	22.53±1.39^{abc}	38.86±1.9^{jkl}	23.4±0.6^{s}	59.05±0.78^{cd}	113.5±0.5^{no}
JG2016-74315	35.34±2.31^{cd}	3.07±0.15^{cdefghij}	27.04±1.81^{ijklmn}	31.37±1.85^{abcdef}	17.94±0.79^{cdefg}	64.83±1.14^{klmnopqr}	110±1.32^{jklm}
JG2016-921814	28.73±0.45^{a}	2.94±0.08^{bcdefghi}	28.5±1.56^{lmno}	30.7±1.78^{abcde}	21.66±0.48^{opq}	61.49±2.38^{efgh}	104.5±1.8^{cdef}
JG2016-9605	37.88±3.59^{cdef}	2.75±0.38^{abcdefg}	25.61±1.23^{defghijkl}	34.88±1.94^{defghij}	20.55±1.01^{lmno}	63.24±0.89^{ghijklm}	107±1^{efghij}
JG2016-9651	29.66±2.15^{ab}	2.90±0.32^{bcdefgh}	23.82±1.67^{abcdefgh}	38.31±2.75^{hijkl}	19.74±1.3^{ijkl}	63.41±1.41^{ghijklm}	106.67±1.89^{defghij}
JG2017-48	29.46±1.25^{ab}	3.56±0.35^{lmn}	25.41±2.39^{cdefghijk}	34.85±2.84^{defghij}	22.55±0.81^{qrs}	62.52±0.9^{fghij}	105±1.32^{cdefg}
JG2018-51	29.2±1.51^{ab}	3.32±0.11hijkl	25.05±1.77^{cdefghijk}	36.29±3.13^{fghijk}	18.28±0.36^{defgh}	66.91±1.69^{pqrs}	102.17±1.04^{abc}
JG2021-1424	36.65±3.46^{cde}	2.65±0.2^{abc}	26.94±2.99^{ijklmn}	30.9±3.16^{abcde}	16.86±0.95^{c}	61.47±1.06^{efgh}	106.67±0.76^{defghij}
JG2021-1617	28.44±2.72^{a}	2.89±0.16^{bcdefgh}	24.71±3.64^{cdefghi}	33.79±5.32^{bcdefghi}	17.66±0.52^{cdef}	56.42±0.58^{ab}	106.5±2.29^{defghi}
JG2021-6301	29.92±2^{ab}	2.91±0.15^{bcdefgh}	25.24±2.01^{cdefghijk}	34.32±3.32^{defghij}	14.06±0.91^{a}	65.92±1.18o^{pqrs}	109.67±1.61^{ijklm}
JG2022-74	30.12±1.37^{ab}	3.17±0.19^{efghijkl}	26.45±1.91^{fghijklm}	33.39±2.56^{bcdefgh}	19.9±0.63^{jklm}	66.99±0.86^{qrs}	107.67±2.36^{fghijkl}
JG2022-75	29.05±0.95^{ab}	3.12±0.3^{defghijkl}	27.57±1.87^{jklmn}	30.21±1.88^{abcd}	18.84±0.41^{fghij}	67.52±0.93^{s}	106.83±1.76^{defghij}
JG226	39.17±2.13^{defg}	2.75±0.07^{abcdefg}	23.73±0.81^{abcdefg}	38.46±1.55^{ijkl}	21.64±0.86^{opq}	61.22±1.21^{efg}	108±1.5^{ghijkl}
JG24	28.02±0.8^{a}	2.73±0.19^{abcdef}	28.74±1.63^{mno}	26.86±3.78^{a}	30.75±0.59^{wx}	67.08±1.13^{rs}	114.83±0.29^{o}
JG28	26.33±1.73^{a}	2.93±0.11^{bcdefghi}	23.47±0.68^{abcde}	30.21±0.63^{abcd}	18.54±0.37^{efghi}	63.69±0.41^{hijklmno}	108.5±0.87^{hijklm}
JG32	41.29±3.12^{fgh}	2.99±0.33^{bcdefghij}	23.27±0.84^{abcde}	37.1±2.73^{ghijkl}	16.78±0.13^{c}	73.08±0.95^{t}	106.83±2.02^{defghij}
JG33	36.58±1.5^{cde}	2.72±0.13^{abcde}	26.11±0.92^{efghijklm}	35.51±0.88^{efghij}	17.4±0.36^{cde}	54.75±0.5^{a}	106.17±1.61^{defgh}

JG36	36.63±1.4^{cde}	3.04±0.27^{cdefghij}	21.44±0.84^{ab}	43.78±3.95^{mn}	16.83±0.74^{c}	62.64±1.18^{fghijk}	113.5±1.73^{no}
JG42	40.86±0.51^{efg}	3.05±0.22^{cdefghij}	24.48±2.3^{cdefghi}	37.85±3.75^{hijkl}	19.1±0.5^{ghijk}	60.58±1.15^{def}	111.67±2.75^{mn}
JG6	45.83±2.02^{i}	3.12±0.05^{defghjikl}	26.66±0.15^{ghijklm}	50.17±0.64^{o}	31.05±0.21^{x}	57.33±0.88^{bc}	104±1.32^{bcde}
JG63	45.3±2.95^{hi}	4.32±0.35^{o}	22.73±0.91^{abcd}	45.85±2.09^{n}	17.75±0.29^{cdef}	65.83±0.63^{nopqrs}	110.67±2.08^{klmn}
JG74	41.36±2.31^{fgh}	2.57±0.27^{ab}	30.53±0.25^{o}	34.12±1.91^{cdefghijk}	17.04±0.49^{cd}	65.72±1.76^{nopqrs}	119.33±1.53^{p}
PG205	45.67±0.5^{hi}	3.93±0.28^{n}	38.08±1.21^{p}	28.58±1.83^{ab}	21.9±0.3^{pqr}	66.8±2.05^{pqrs}	107.5±1.8^{fghijk}
**Mean**	**36.24±9.01**	**3.25±0.59**	**26.37±3.91**	**35.25±5.32**	**20.83±4.21**	**62.87±3.86**	**107.35±4.06**

Data pooled for two successive seasons and presented as mean of triplicate ± standard deviation. Means with the same letter are not significantly different at 5% level of significance

Supplementary Table S7 Pooled yield and its attributing trait responses of various chickpea genotypes under drought stressat vegetative stage

**Genotypes**	**DTF**	**DTM**	**NOP**	**SY**	**BY**	**HI**	**100SW**
ICC4958	49.95±0.64^{a}	77.17±1.04^{a}	28.47±2.52^{k}	3.53±0.23^{klmn}	12.51±0.68^{klmn}	28.18±0.34^{defghij}	29.49±0.53^{o}
ICCV15102	58.71±0.33^{mnopqr}	95.44±0.45^{pqr}	13.35±1^{b}	2.66±0.11^{abcd}	10.26±0.27^{abcd}	25.93±0.46^{bcdefg}	23.26±0.36^{l}
ICCV15115	54.94±0.9^{defgh}	90.94±1.09^{hijkl}	18.02±1.04^{cde}	3.38±0.1^{ijkl}	11.75±0.5^{ijkl}	28.72±2.04^{defghij}	21.95±0.89^{ijkl}
ICCV15118	55.99±0.79^{fghijk}	90.65±0.81^{hijkl}	18.85±0.5^{cdefg}	3.35±0.09^{hijkl}	11.86±0.86^{hijkl}	28.47±2.4^{defghij}	19.95±1.26^{fg}
ICCV181664	58.76±1.06^{nopqr}	92.23±0.13^{klmn}	16.85±2.29^{bcd}	3.14±0.21^{efghijkl}	8.73±0.3^{efghijkl}	35.98±1.12^{l}	20.4±1.02^{fgh}
ICCV19616	57.79±1.55^{jklmnop}	92.25±0.06^{klmn}	18.68±2.57^{cdefg}	3.88±0.09^{n}	11.91±0.12^{n}	32.66±0.41^{ijkl}	22.88±0.95^{kl}
JAKI9218	54.07±0.52^{cdef}	83.63±1.42^{b}	32.47±3.4^{lm}	4.70±0.37^{op}	15.30±0.86^{op}	30.78±1.81^{ghijk}	27.75±0.24^{n}
JG11	51.6±1.01^{ab}	86.63±0.6^{de}	26.63±2.31^{jk}	4.82±0.35^{p}	14.59±0.75^{p}	33.04±1.31^{jkl}	25.04±0.09^{m}
JG14	55.19±0.52^{defghi}	87.13±1.15^{def}	13.3±0.5^{b}	2.32±0.06^{a}	9.20±0.42^{a}	25.4±0.42^{bcdef}	19.32±0.74^{ef}
JG16	54.52±0.87^{defg}	95.69±1.16^{qr}	33.3±0.5^{m}	4.34±0.06^{o}	19.28±0.89^{o}	22.58±1^{ab}	25.6±0.38^{m}
JG17	53.9±0.54^{cde}	89.81±2.49^{ghijk}	22.8±1.32^{fghij}	3.11±0.14^{cdefghijk}	11.03±1.49^{cdefghijk}	28.4±2.59^{defghij}	16.94±0.59^{cd}
JG2003-14-16	54.58±0.58^{defg}	85.94±0.63^{cd}	24.47±3.55^{ij}	3.23±0.14^{hijkl}	10.51±1.64^{hijkl}	31.08±3.66^{hijkl}	22.73±0.85^{jkl}
JG2016-1411	53.66±0.24^{cd}	90.69±2.01^{hijkl}	22.47±1.76^{fghi}	3.20±0.31^{ghijkl}	9.35±0.39^{ghijkl}	34.24±2.44^{kl}	17.36±1.67^{d}
JG2016-1614	59.36±1.61^{opqrs}	88.48±0.41^{efgh}	22.3±2.5^{efghi}	3.19±0.16^{ghijkl}	11.45±0.69^{ghijkl}	27.9±0.32^{cdefghi}	14.71±1.11^{b}
JG2016-36	59.39±0.54^{opqrs}	95.27±0.78^{pqr}	26.8±2.18^{jk}	3.45±0.23^{jklm}	13.72±0.99^{jklm}	25.25±2.67^{bcdef}	16.03±0.24^{bc}
JG2016-44	58.12±0.57^{lmnopq}	91.63±0.6^{ijklmn}	22.97±1.44^{ghij}	3.06±0.25^{cdefghij}	10.87±1.08^{cderfghij}	28.19±1.47^{defghij}	17.71±0.55^{d}
JG2016-45	56.73±0.81^{hijklm}	90.77±1.75^{hijkl}	18.8±3.61^{cdefg}	3.82±0.36^{lmn}	14.87±0.37^{lmn}	25.66±1.79^{bcdefg}	17.9±0.64^{d}
JG2016-634958	53.31±0.9^{bcd}	93.94±1.66^{nopqr}	21.97±2.75^{efghi}	3.19±0.35^{fghijkl}	9.81±0.6^{fghijkl}	32.51±3.88^{ijkl}	22.27±0.52^{jkl}
JG2016-74315	57.89±1.2^{klmnop}	92.75±1.06^{lmno}	21.13±0.76^{defghi}	3.07±0.15^{cdefghij}	10.68±0.34^{cdefghij}	28.67±0.53^{defghij}	17.53±0.88^{d}
JG2016-921814	56.64±2.38^{hijkl}	89.44±1.08^{fghi}	18.63±1.15^{cdef}	2.94±0.08^{bcdefghi}	10.93±0.17^{bcdefghi}	26.89±0.47^{bcdefgh}	21.57±0.77^{ijkl}
JG2016-9605	56.06±0.81^{fghijk}	91.25±1.11^{ijklm}	21.8±3.04^{efghi}	2.75±0.38^{abcdefg}	10.65±1.39^{abcdefg}	26.53±5.25^{bcdefgh}	19.66±0.4^{fg}
JG2016-9651	56.74±1.65^{hijklm}	92.67±1.32^{lmno}	19.97±1.26^{cdefgh}	2.90±0.32^{bcdefgh}	9.98±0.13^{bcdefgh}	29.12±3.43^{efghij}	19.18±1.06^{ef}
JG2017-48	57.06±0.16^{ijklmn}	90.88±0.92^{hijkl}	19.63±2.47^{cdefgh}	3.56±0.35^{lmn}	14.66±0.94^{lmn}	24.28±0.93^{abcde}	22.58±0.89^{jkl}
JG2018-51	61.2±0.65st	87.98±1.23^{defg}	23.47±0.76^{hij}	3.32±0.11hijkl	11.14±2.23^{hijkl}	30.58±5.65^{ghijk}	17.68±0.55^{d}
JG2021-1424	56.14±0.41^{ghijkl}	93.04±0.73^{lmnop}	18.13±1.89^{cde}	2.65±0.2^{abc}	9.40±0.62^{abc}	28.25±1.99^{defghij}	15.98±0.92^{bc}
JG2021-1617	50.5±0.4^{a}	92.57±2.78^{lmno}	20.13±1.61^{cdefgh}	2.89±0.16^{bcdefgh}	9.80±0.46^{bcdefgh}	29.5±2.98^{fghijk}	17.1±0.35^{cd}
JG2021-6301	60.2±1.23^{rst}	94.92±0.38^{opqr}	19.63±1.53^{cdefgh}	2.91±0.15^{bcdefgh}	10.18±2.06^{bcdefgh}	29.14±4.57^{defghij}	13.5±1.07^{a}
JG2022-74	59.66±0.66^{pqrs}	93.67±2.54^{mnopq}	22.97±2.02^{ghij}	3.17±0.19^{efghijkl}	14.06±1.6^{efghijkl}	22.64±1.27^{ab}	19.73±0.5^{fg}
JG2022-75	61.88±0.72^{t}	92.77±1.47^{lmno}	19.47±3.18^{cdefgh}	3.12±0.3^{defghijkl}	13.18±0.53^{defghijkl}	23.69±1.96^{abcd}	18.06±0.5^{de}
JG226	55.78±1.07^{efghij}	91.75±1.7^{ijklmn}	8.49±1.63^{a}	2.75±0.07^{abcdefg}	10.12±0.38^{abcdefg}	27.2±1.06^{bcdefgh}	20.94±0.87^{ghi}
JG24	57.11±1.16^{ijklmn}	98.02±1.15^{s}	13.8±1.8^{b}	2.73±0.19^{abcdef}	11.96±0.89^{abcdef}	22.92±2.1^{abc}	29.37±0.35^{o}
JG28	54.98±1.66^{defgh}	92.06±0.29^{jklmn}	19.8±0.87^{cdefgh}	2.93±0.11^{bcdefghi}	10.53±0.59^{bcdefghi}	27.88±0.58^{cdefghi}	17.54±0.49^{d}
JG32	66.79±0.97^{u}	89.65±0.47^{ghij}	19.97±3.21^{cdefgh}	2.99±0.33^{bcdefghij}	9.11±0.62^{bcdefghij}	32.97±4.95^{ijkl}	15.56±0.44^{b}
JG33	50.54±0.67^{a}	89.35±0.97^{fghi}	16.8±1.32^{bc}	2.72±0.13^{abcde}	8.64±0.34^{abcde}	31.61±2.75^{hijkl}	15.07±0.48^{b}

JG36	56.3±1.54^{ghijkl}	93.94±0.58^{nopqr}	25.13±2.89^{ijk}	3.04±0.27^{cdefghij}	11.73±0.41^{cdefghij}	25.92±2.64^{bcdefg}	15.57±0.34^{b}
JG42	55.07±0.57^{defghi}	96.29±2.49^{rs}	19.47±1.76^{cdefgh}	3.05±0.22^{cdefghij}	8.96±0.44^{cdefghij}	34.22±4.06^{kl}	18.27±0.6^{de}
JG6	52.52±0.57^{bc}	83.87±1.55^{bc}	17.13±3.33^{bcd}	3.12±0.05^{defghjikl}	9.96±0.31^{defghijkl}	31.53±0.4^{hijkl}	30.07±0.43^{o}
JG63	60.06±1.28^{qrst}	95.79±0.79^{qrs}	28.97±1.76^{kl}	4.32±0.35^{o}	10.75±1.38^{o}	40.42±1.83^{m}	17.18±0.33^{cd}
JG74	57.62±1.29^{jklmno}	104.09±0.89^{t}	13.4±1.07^{b}	2.57±0.27^{ab}	9.47±0.36^{ab}	27.19±3.01^{bcdefgh}	15.65±0.39^{b}
PG205	60.63±1.99^{rst}	93.69±1.47^{mnopq}	23.3±3.12^{hij}	3.93±0.28^{n}	19.61±1^{n}	20.08±2.23^{a}	21.45±0.29^{ijk}
**Mean**	**56.55±3.47**	**91.47±4.54**	**20.84±5.34**	**3.25±0.59**	**11.56±2.64**	**28.65±4.53**	**20.01±4.23**

Data pooled for two successive seasons and presented as mean of triplicate ± standard deviation. Means with the same letter are not significantly different at 5% level of significance
